# Efficient delivery of methotrexate to MDA-MB-231 breast cancer cells by a pH-responsive ZnO nanocarrier

**DOI:** 10.1038/s41598-023-49464-9

**Published:** 2023-12-11

**Authors:** Jiko Raut, Olivia Sarkar, Tanmoy Das, Santi M. Mandal, Ansuman Chattopadhyay, Prithidipa Sahoo

**Affiliations:** 1 Department of Chemistry, Visva-Bharati University, Santiniketan, 731235 India; 2 Department of Zoology, Visva-Bharati University, Santiniketan, 731235 India; 3 Central Research Facility, Indian Institute of Technology Kharagpur, Kharagpur, 721302 India

**Keywords:** Drug delivery, Cancer

## Abstract

Methotrexate (MTX), an efficient chemotherapy medication is used in treating various malignancies. However, the breast cancer cell line MDA-MB-231 has developed resistance to it due to low levels of the MTX transport protein, and reduced folate carrier (RFC), making it less effective against these cancer cells. Here we designed a very simple, biocompatible, and non-toxic amine-capped ZnO quantum dots to overcome the MTX resistance on the MDA-MB-231 breast cancer cell line. The QD was characterized by HRTEM, DLS EDX, FT-IR, UV–Vis, and Fluorescence spectroscopy. MTX loading onto the QD was confirmed through fluorescence and UV–Vis spectroscopy. Additionally, extensive confocal microscopic investigations were carried out to determine whether the MTX was successfully released on the MDA-MB-231 cell line. It was discovered that QD is a better pH-responsive delivery system than the previous ones because it successfully delivers MTX to the MDA-MB-231 at a higher rate on an acidic pH than it does at a physiological pH. QD also has anticancer activity and can eradicate cancer cells on its own. These factors make the QD to be an effective pH-responsive delivery system that can improve the efficacy of the medication in therapeutic diagnosis.

## Introduction

The development of innovative treatments for cancer is the main focus to significantly prolong and ensure a good quality of life for cancer patients. Hence, nowadays, one of the most pressing global concerns is the progress of new and cutting-edge cancer treatments. Novel ideas and techniques are effectively being conducted every day to treat and eradicate cancer. One of the traditional and often utilized cancer treatment options is chemotherapy. However, repeated exposure to chemotherapeutic drugs speeds up the emergence of severe acquired resistance, particularly multidrug resistance (MDR)^1^. Drug resistance results in a number of mechanisms, such as increased drug efflux, growth factors, genetic factors (epigenetic changes, amplifications, and mutations) improved DNA repair capacity and heightened xenobiotic metabolism^2^, which decrease the efficacy of antineoplastic agents. Thus, the effect on patient survival would be greater if drug resistance could be reduced.

Presently, nanomedicines are being used as a leading tool in drug delivery systems. However, due to the inadequate stability, selectivity, solubility, and quick body clearance, nanoparticle-based delivery systems, such as proteins, liposomes, polymers, inorganic/organic hybrid materials, and Quantum Dots have been advanced for selective delivery of anti-tumor medications^3^. Methotrexate (MTX) is a folate antagonist^4^ and antineoplastic drug that is frequently used for cancer chemotherapy as well as the treatment of psoriasis, rheumatoid arthritis, and autoimmune diseases as well as for the prevention of graft-versus-host disease after transplantation^5^. In order to prevent efflux, MTX enters the cells through RFC and takes place polyglutamylation through folylpolyglutamate synthetase (FPGS)^6^. It acts to produce tetrahydrofolate from dihydrofolate by inhibiting dihydrofolate reductase (DHFR), an essential enzyme for intracellular folate metabolism. Consequences of DHFR inhibition include the impediment to tetrahydrofolate synthesis from dihydrofolate and the accumulation of dihydrofolate polyglutamate within the cell causing impairment of thymidylate and purine biosynthesis, which in turn slows down DNA replication and speeds up apoptosis^5,7^. To the contrary, MTX exhibits MDR via a number of mechanisms, including, dihydrofolate reductase gene amplification, decreased folylpolyglutamate synthetase activity, decreased folate carrier-mediated membrane transport, and enhanced MTX outflow from the cell through elevating folylpolyglutamate hydrolase activity^4,6^.

Numerous strategies have been devised by researchers to combat methotrexate resistance in cancer cells^8–13^. For intercellular distribution, Lindgren's team created a cell-penetrating peptide (CPP) linked with MTX^4^. However, the covalent construction of the CPP-drug delivery system, in which CPP and its payload are joined by a covalent bond, may result in subpar drug release^14^. To circumvent the resistance, Wu and colleagues created the methotrexate prodrug^15^. Despite being a successful strategy for combating drug resistance, prodrug frequently causes subpar drug release from the covalently coupled MTX. Currently, several researchers are interested in using nano-carriers as drug delivery systems (DDDs). MTX-conjugated quantum dots (QDs) have been introduced as drug delivery systems^16^. QDs are semiconductor nanocrystals with distinctive electrical and optical characteristics that have a lot of potential for the creation of theragnostic^17–24^. Since cadmium is the core component of most MTX-conjugated QDs, even though they have various applications, are restricted by their toxicity. Therefore, it is important to create a drug delivery arrangement with low toxicity and quick drug release. According to reports, ZnO QDs, a novel class of economical and low-toxic nanomaterial, perform better in biological applications^25,26^. The modern ZnO QDs drug delivery methods have many benefits over conventional drug delivery methods. ZnO QDs are perfect for biological applications because they are non-toxic and barely harmful. ZnO QDs have been reported to be inexpensive, simple to make and to react to acid. At pH < 5.5, ZnO QDs have the capacity to dissolve quickly to Zn^2+^^27^. Furthermore, research on the behavioral changes of ZnO QDs during dissolution revealed that these particles only showed cytotoxic effects that were considerable after dissolution and that they preferred to kill cancer cells^28,29^. Given all of the above-mentioned excellent ZnO QD features, the pH-sensitive ZnO QDs drug delivery system was expected to demonstrate significantly higher therapeutic efficacy^15,16^. Therefore, in our study, we designed a ZnO QD with a surface modified by an amine (diethylenetriamine). This amine-capped ZnO QD is inexpensive, non-toxic, and suitable for biological applications. The amine-capped ZnO QD interacts with MTX via a non-covalent interaction, which greatly enhances the drug release. In our study, we choose MDA-MB-231, a triple-negative breast cancer cell line, a methotrexate-resistant cancer cell due to low levels of MTX transport protein, and RFC^14^. As far as we are aware, this is the first quantum dot used as a nanocarrier that delivers MTX to its resistant cancer cell MDA-MB-231 by non-covalent interaction even without using any other prodrug or via covalent conjugation. Here, pH functions as a natural stimulant for the dissolving of QDs at various pH values, leading to various drug release behaviors. It is known that DDSs respond to pH in order to release a specific drug. These systems chemical composition or physical structure changes, degrades, or dissolves when certain triggers act on them, causing a controlled release of drugs at the targeted site. The tumor microenvironment differs from normal cells in that it has unique properties including a lower pH (5.5–6.7) and a greater temperature, which can be used to create internal stimuli-responsive (e.g., pH) drug delivery systems. In a basic condition (typically in PBS buffer, pH 7.4), the primary reason for the disintegration of QDs is the replacement of their organic ligands by phosphate ions. This is because in an acidic condition, proton and metal ions compete for coordination with the organic ligand. The protonation of the QD surface ligand or drug, which has a substantial impact on the drug release kinetics, is caused by additional factors such as altered interactions between the drug molecules at various pH values^30,31^. The study shows that our design system delivers the drug into the targeted cancer cell more efficiently at acidic pH than at physiological pH and also demonstrated significant antitumor activity of the QD itself, implying that the combination of the drug with the quantum dots showed synergistic cytotoxicity against breast cancer cells. These benefits make a practical and distinctive drug delivery method that is based on pH-responsive quantum dots and can improve the therapeutic outcome of chemotherapy.

## Results and discussions

### Morphological analysis

**Figure 1:**
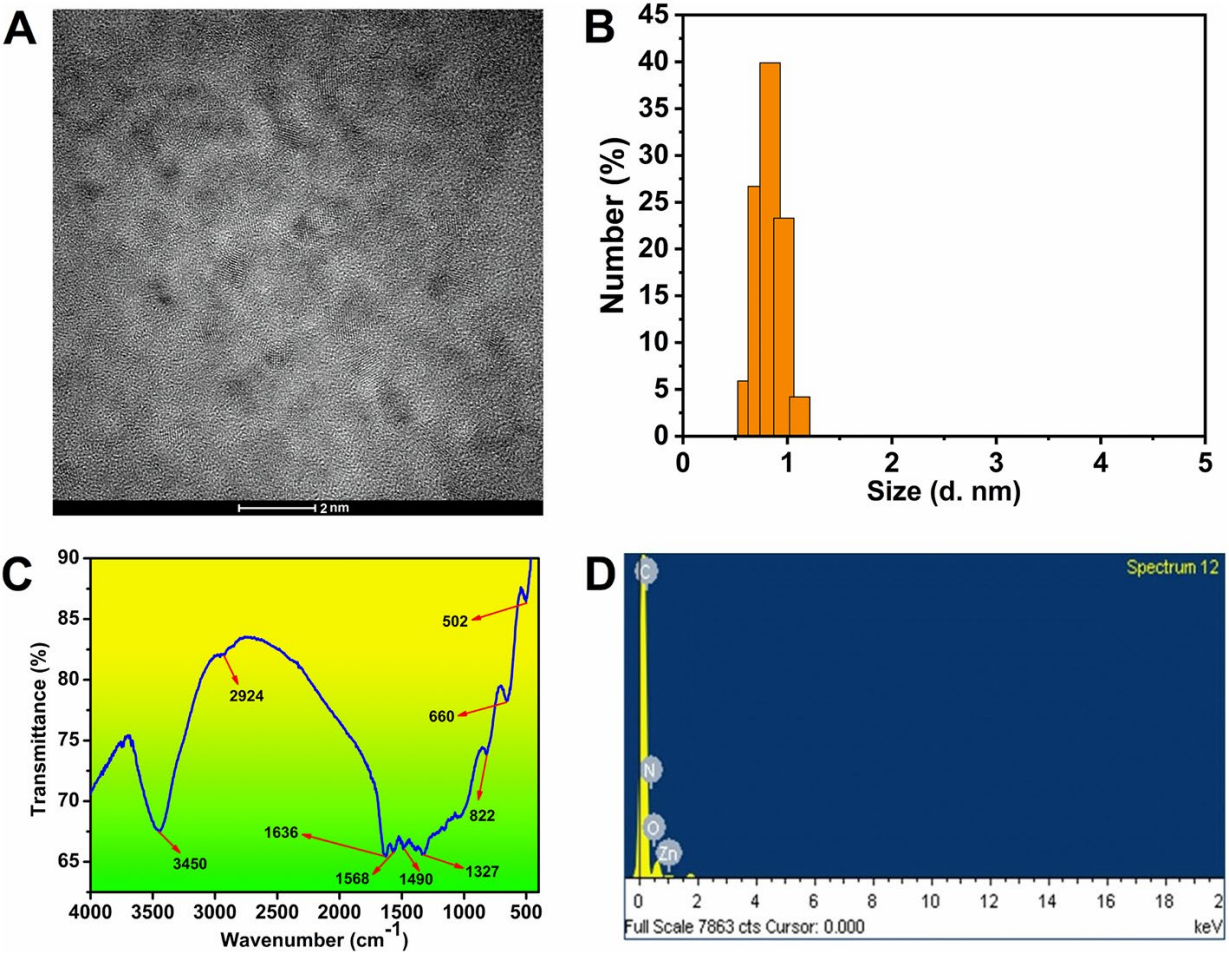
(**A**) HRTEM image of the QD (**B**) DLS data (**C**) FT-IR spectra and (**D**) EDX data of QD.

According to the results of the HRTEM image (Fig. 1A) and DLS experiment (PDI = 0.5) (Fig. 1B), the produced QDs are monodispersed and range in size from 0.5 to 1 nm with clearly visible lattice fringe, which further demonstrates the crystalline nature of the QDs.

### FT-IR analysis

FT-IR spectra utilized to assess the QDs surface functional group are shown in Fig. 1C. Stretching vibrations of -NH and C-H brought the peaks at 3450 cm^−1^ and 2924 cm^−1^, respectively. The bending vibrations of –C=O–NH– are shown in the peaks at 1568 cm^−1^ and 1636 cm^−1^. The nitrogen doping has been confirmed by 1490 cm^−1^ and 1327 cm^−1^ peaks which are attributed to C–N–C and C–N, respectively. The peak at 502 cm^−1^ represents the Zn–O bond^32–36^. The zeta potential of the QD was found to be − 7.45 mV.

The element composition of QDs was studied using energy-dispersive X-ray spectroscopy (EDX) (Fig. 1D), which demonstrated the presence of C, N, O, and Zn atoms.

### Optical properties of amine (DETA) capped ZnO QD

**Figure 2:**
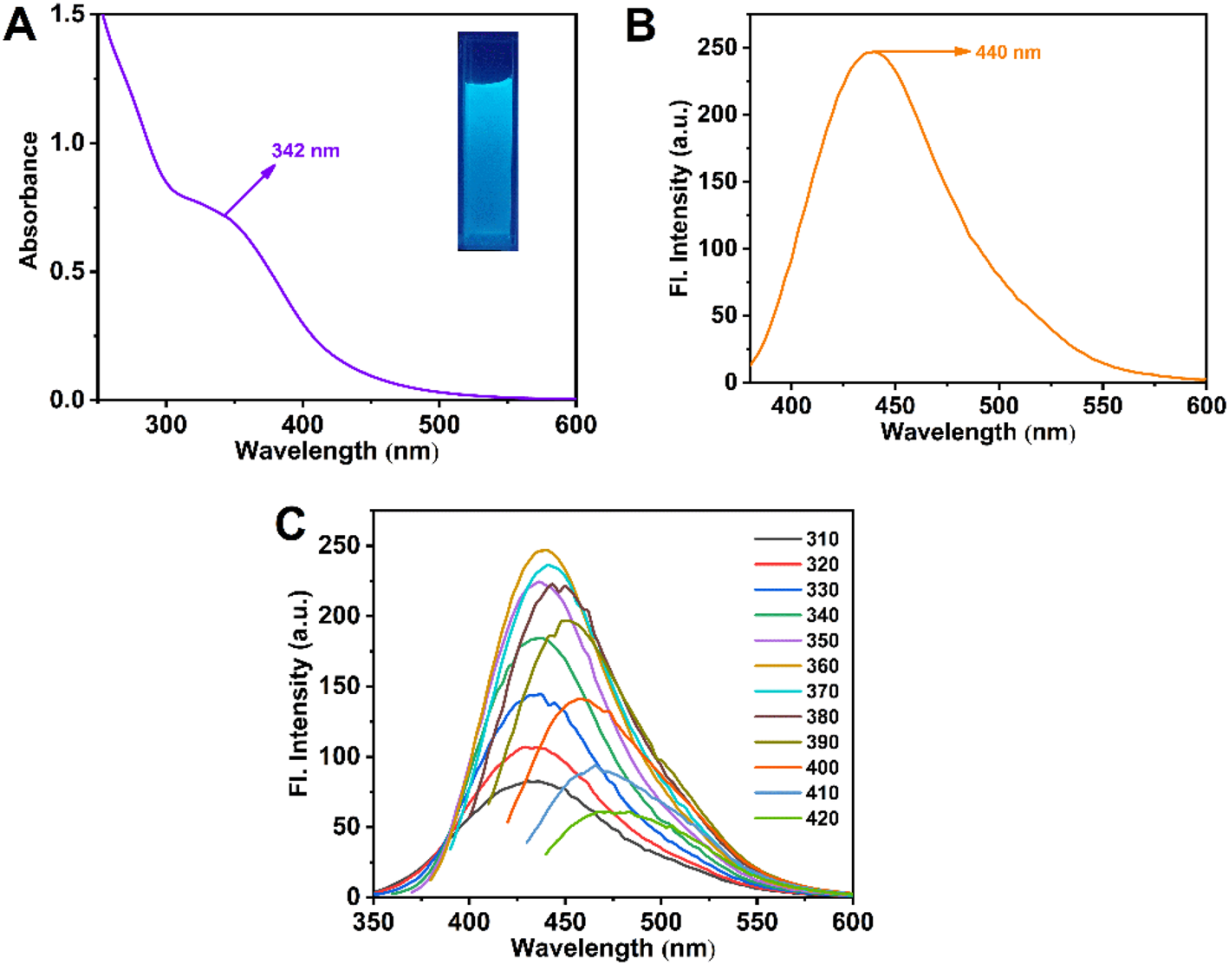
Spectroscopic data of QD: (**A**) UV–vis spectra (**B**) Fluorescence emission spectrum (**C**) Emission spectra at various excitation.

The UV–Vis and fluorescence spectra of the QDs are presented in Fig. 2A,B, respectively. An absorption band at 342 nm is represented in the UV–Vis spectra of QD due to the n-π* transition^37–39^. Moreover, under UV light (365 nm), QD shows a strong blue fluorescence (inset in Fig. 2A). QD exhibits a significant 440 nm emission peak after being excited at 360 nm. Figure 2C displays the excitation-dependent fluorescence behavior of the QD. Upon excitation from 310 to 360 nm, the fluorescence intensity increases and then declines from 370 to 420 nm excitation and red-shifted. The intensity of the fluorescence emission is highest at 440 nm when excited at 360 nm.

**Figure 3:**
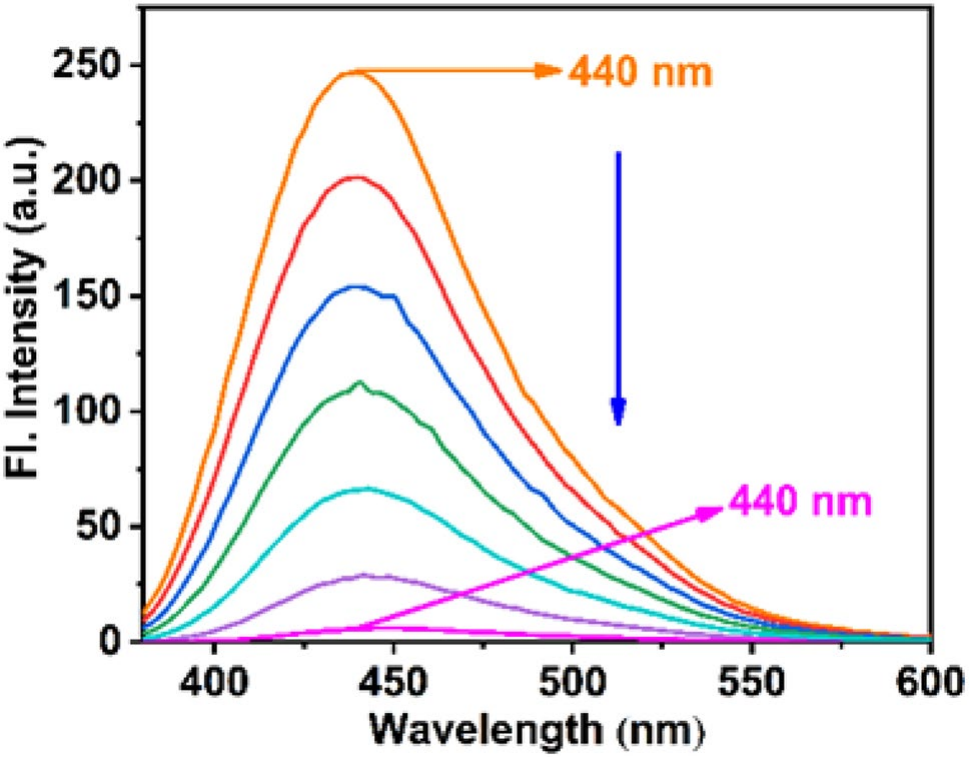
Fluorescence of QD quenched by the addition of different concentration of MTX (10^–3^ M).

Now to confirm the drug loading into our nanocarrier we performed fluorescence and UV–vis spectroscopy. From Fig. 3 we can see that after the addition of MTX (10^–3^ M) into the QD solution there is a significant fluorescence quenching observed which conclusively proved that MTX is successfully loaded into the QD surface. It was estimated that the MTX loading on amine-capped ZnO QDs could reach 25%. Regression analysis was used to calculate the binding affinity of the quantum dots for MTX, which was found to be 4.8 × 10^5^ M^-1^ (Figure S1). Further UV–Vis spectra also corroborated the same results as fluorescence (Figure S2).

### Possible quenching mechanism

**Figure 4:**
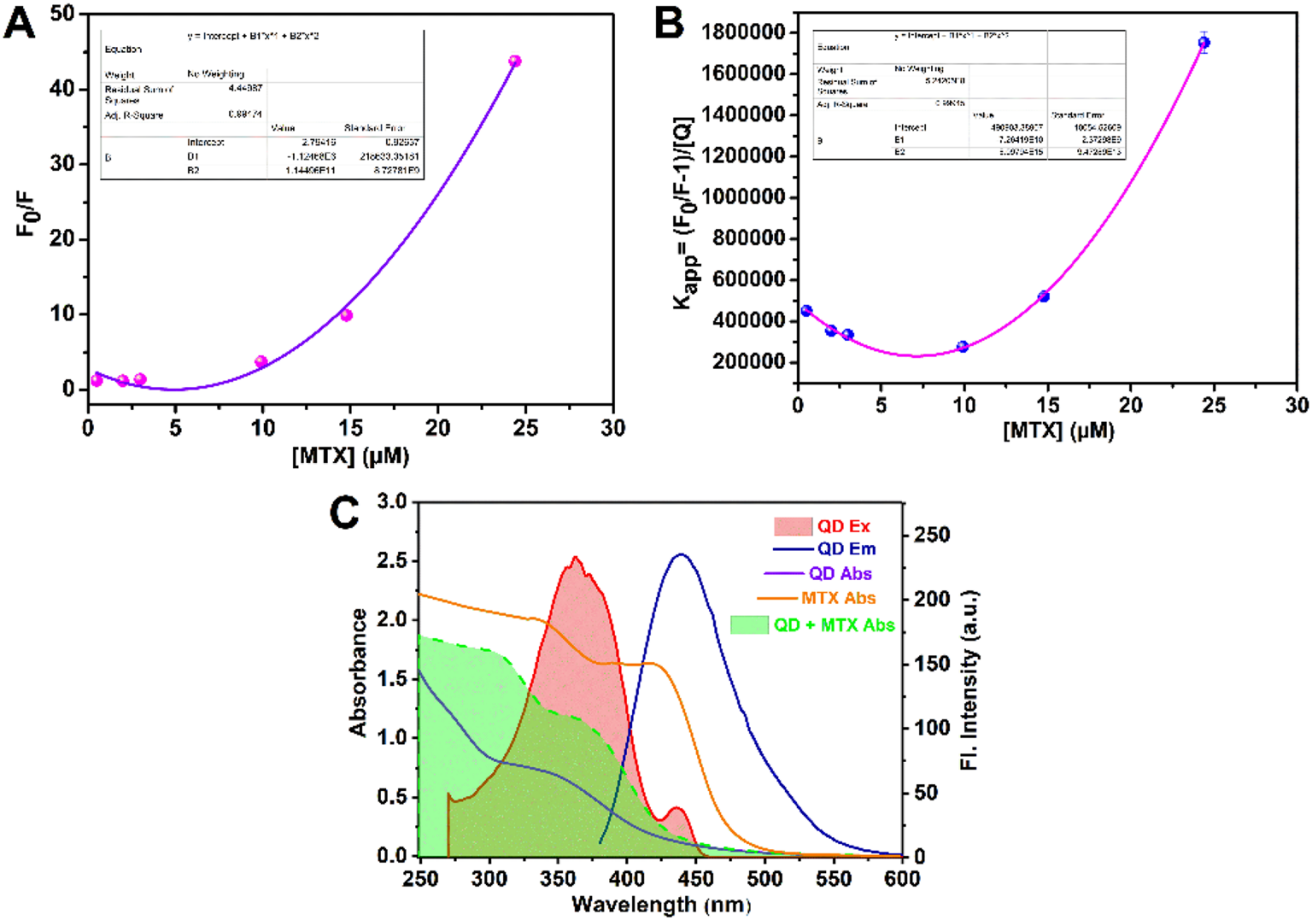
(**A**) polynomial fitting (second-order) of Stern–Volmer plot for MTX (**B**) Kapp Vs concentration plot of MTX (**C**) Excitation & emission spectra of QD indicated in red gradient & blue line, respectively, absorption of QD (violet line), MTX (orange line) & QD + MTX (green gradient).

We hypothesized that the fluorescence quenching is due to the sphere of action mechanism. To examine if such type of quenching has occurred, we used the Stern–Volmer Eq.^40^. The type of quenching determines whether a linear or non-linear Stern–Volmer plot is obtained. Three distinct types of quenching are possible a) dynamic quenching b) static quenching and c) combined dynamic and static quenching. Plotting F_0_/F Vs [Q] produces a linear curve in cases of static and dynamic quenching, which is not observed in our investigation. F_0_ and F are the fluorescence intensities without and with the quencher (MTX), respectively, and Q is the concentration of the quencher (MTX). An upward (positive) curvature should be obtained in circumstances of simultaneous dynamic and static quenching, and in our case, this is what we found (Fig. 4A). Hence, fluorescence quenching may result from the combination of static and dynamic quenching. Fluorescence is suppressed simultaneously by two mechanisms when dynamic and static quenching are combined: first, dynamic quenching, and then static quenching by producing a ground-state nonfluorescent complex. At first, to confirm the involvement of dynamic quenching we used UV–vis spectroscopy. Dynamic quenching affects QDs excited states rather than their ground state. Hence, it is unlikely that the absorbance spectra will alter during dynamic quenching. Changes in the absorbance spectrum of the QDs are expected in static quenching when a ground state complex is formed which is clearly observed in our case as absorption spectra shift from 342 nm to 378 after interaction with MTX (Figure S2). So, the involvement of dynamic quenching is rejected. Now to confirm the static quenching by the formation of a nonfluorescent complex in the ground state the modified Stern–Volmer equation is used-

1 $$ {\text{K}}_{{{\text{app}}}} = \, \left( {{\text{F}}_{0} /{\text{F}}{-}{1}} \right)/\left[ {\text{Q}} \right]) \, = \, \left( {{\text{K}}_{{\text{S}}} + {\text{ K}}_{{\text{D}}} } \right) \, + {\text{ K}}_{{\text{S}}} {\text{K}}_{{\text{D}}} \left[ {\text{Q}} \right] $$

If a straight line is obtained by plotting (F_0_/F−1)/[Q]) Vs [Q], quenching is most likely involved by the production of a complex in the ground state, which is not evident here. In this instance, it is evident that the straight line has been deviated (Fig. 4B). Therefore, the idea of mixed dynamic and static quenching is unacceptable. There is another process, which is also responsible for fluorescence quenching referred to as the “sphere of action”. When the quencher is in close proximity to the QD at the time of excitation, fluorescence is quenched without producing a complex in the ground state. This type of quenching is denoted as the “sphere of action” and the Stern–Volmer equation for this is—

2 $$ {\text{F}}_{0} /{\text{F }} = \, \left( {{1} + {\text{K}}_{{\text{D}}} \left[ {\text{Q}} \right]} \right){\text{ e}}^{{{\text{V}}[{\text{Q}}]}} $$

Here, V is the volume of the sphere. plotting F_0_/F Vs [Q] gives a positive upward curve.

Now, as the notion of simultaneous dynamic and static quenching is discarded, however, we still observe an upward positive curve in our case, allowing us to predict that the "sphere of action" is what causes the quenching of the QD^41^.

Moreover, as depicted in Fig. 4C, the absorption spectra of QD with MTX coincide with the excitation spectra of QD without MTX. Hence, the Inner Filter Effect (IFE) is the reason for fluorescence quenching. We can therefore deduce that the inner filter effect (IFE) and the sphere of action both contribute to the fluorescence quenching of QD by MTX^42^.

### Biological study: confocal laser scanning microscopy (CLSM) tests

Now to determine the efficacy of the QD as a nanocarrier to overcome MTX resistance, the confocal microscopic study was conducted.

### MDA-MB-231 cells treated with quantum dot and drug at physiological pH = 7.4

**Figure 5:**
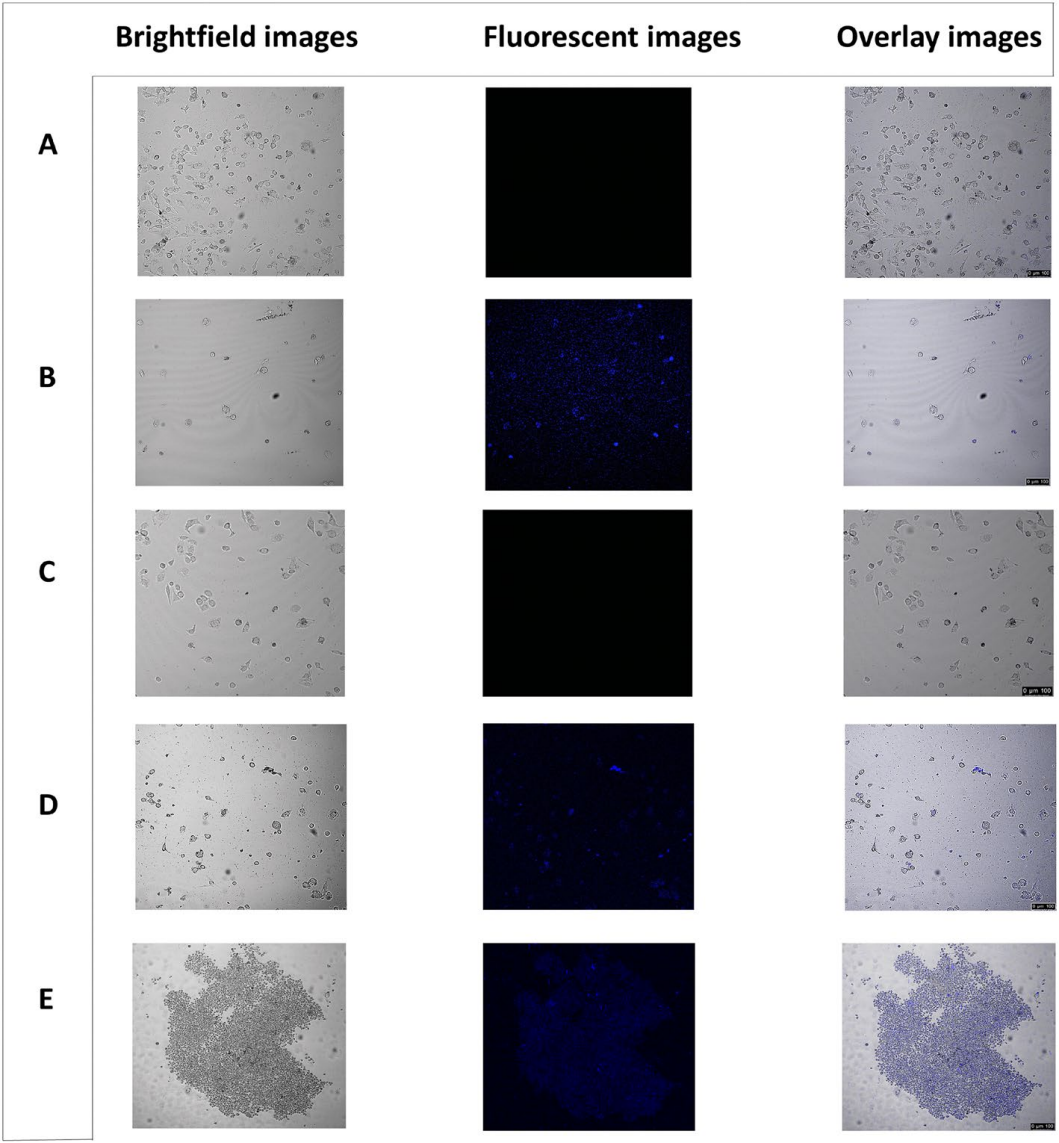
Confocal microscopy images of MDA-MB-231 cells after treatment with quantum dot and drug at pH = 7.4. (**A**) control untreated cells; (**B**) cells treated with 1/3rd IC50 value of quantum dot i.e., 18 ppm (**C**) cells treated with the drug and 18 ppm of quantum dot for 1 day; (**D**) cells treated with the drug and 18 ppm of quantum dot for 3 days; (**E**) cells treated with the drug and 18 ppm of quantum dot for 5 days. Brightfield images, fluorescent images, and overlay images are shown in the left, middle, and right panels respectively. Magnification: 200X. Zoom factor: 1.00. Scale bars are in the range of 0–100 µm.

Biolabeling with the quantum dot and the intracellular release of the drug from the quantum dot in the treated MDA-MB-231 cells was demonstrated by confocal laser scanning microscopy (CLSM). Herein, the fluorescence of the quantum dot was first used to observe the positive endocytosis of the quantum dot. The untreated control cells i.e., the cells treated neither with the quantum dot nor the drug did not exhibit any fluorescence (Fig. 5A). The blue fluorescence in the cytoplasm of the cells treated only with the quantum dot for 1 day clearly indicated the successful penetration of the quantum dots into the cells through endocytosis (Fig. 5B). The blue fluorescence in MDA-MB-231 cells treated with both the quantum dot and the drug demonstrated the release of drug from the quantum dots into the cytoplasm due to the disintegration of the quantum dots in the intracellular acidic compartments of endosomes and lysosomes (pH = 4.5–6.5)^43^. But the intensity of the fluorescence varied with the duration of the treatment of the cells with the quantum dot and the drug. After incubation with a loaded drug, the intense blue fluorescence signal of the quantum dot was diminished in the cytoplasmic region of the cells (Fig. 5C). However, from the 1st day to the 3rd day, there was a gradual increase in the blue fluorescence in the cytosol of the treated cells (Fig. 5D) and finally, at the 5th day, the intensity of the blue fluorescence was the maximum and the treated MDA-MB-231 cells had taken up a clumped appearance (Fig. 5E) suggesting the huge release of the drug from the quantum dots.

### MDA-MB-231 cells treated with quantum dot and drug at acidic pH = 3.5

**Figure 6:**
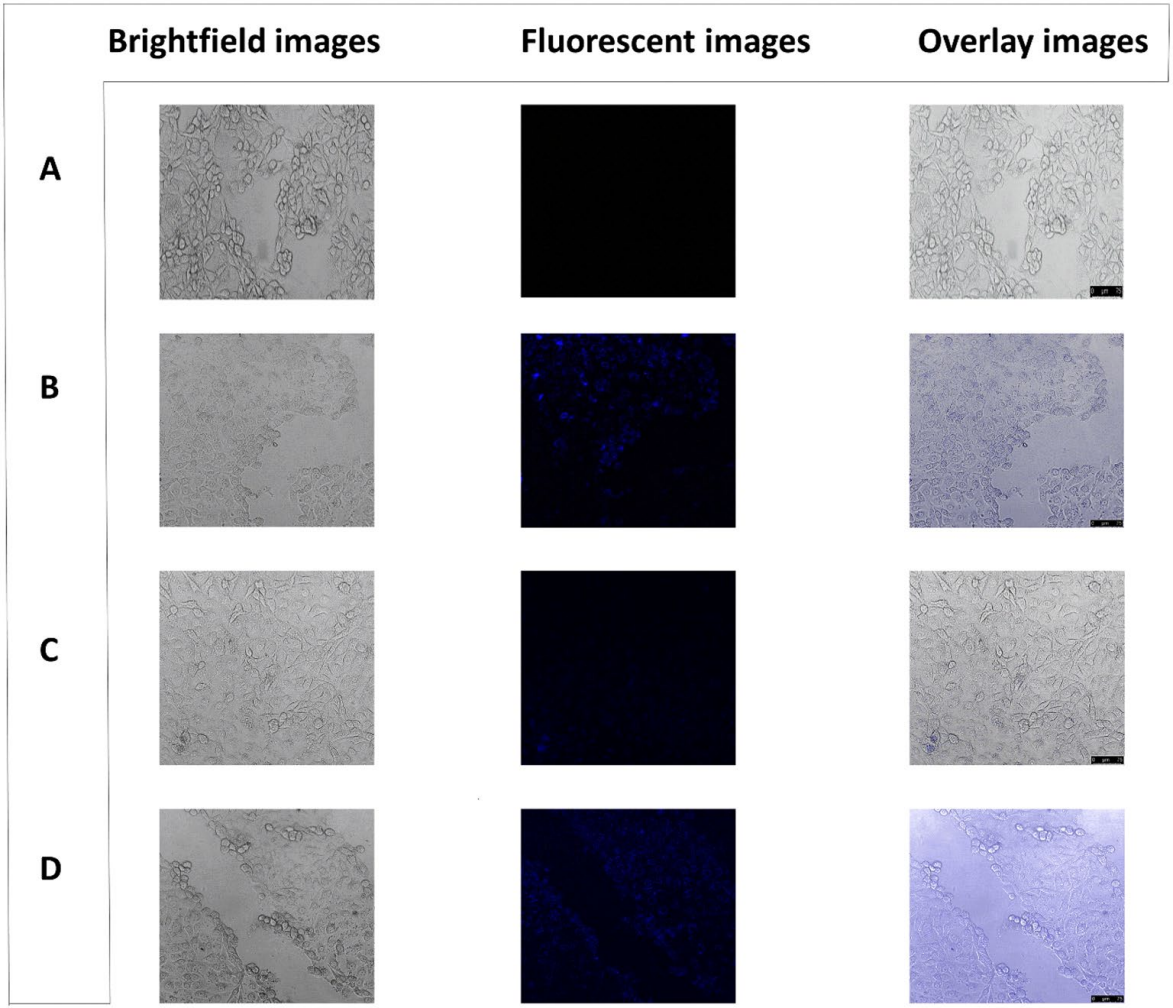
Confocal microscopy images of MDA-MB-231 cells after treatment with quantum dot and drug at pH = 3.5. (**A**) control untreated cells; (**B**) cells treated with 1/3rd IC50 value of quantum dot i.e., 18 ppm (**C**) cells treated with the drug and 18 ppm of quantum dot for 1 day; (**D**) cells treated with the drug and 18 ppm of quantum dot for 2 days. Brightfield images, fluorescent images, and overlay images are shown in the left, middle, and right panels respectively. Magnification: 200X. Zoom factor: 1.00. Scale bars are in the range of 0–75 µm.

The whole experimental set-up was carried out with MDA-MB-231 cells at an acidic pH to check the role of pH in the disassociation of the drug from the quantum dot. No blue fluorescence was detected in the untreated control cells (Fig. 6A). Blue fluorescence was detected in the MDA-MB-231 cells treated with only the quantum dot as the dot itself is fluorescent and could easily penetrate into the cells (Fig. 6B). The cytoplasm of the cells treated with both the quantum dot and the drug emitted blue fluorescence and it established the release of the drug from the quantum dots into the cytoplasm due to the dissolution of the quantum dots (Fig. 6C,D). However, here, the intensity of the fluorescence showed a directly proportional relation to the duration of the treatment of the cells with the quantum dot and the drug. In contrast, the intensity was the highest in the cells treated with the dot and drug for 2 days (Fig. 6D). Here, all the cells were clumped in appearance which indicated the complete dissociation of the drug from the quantum dot.

### HepG2 cells treated with quantum dot and drug at physiological pH = 7.4

**Figure 7:**
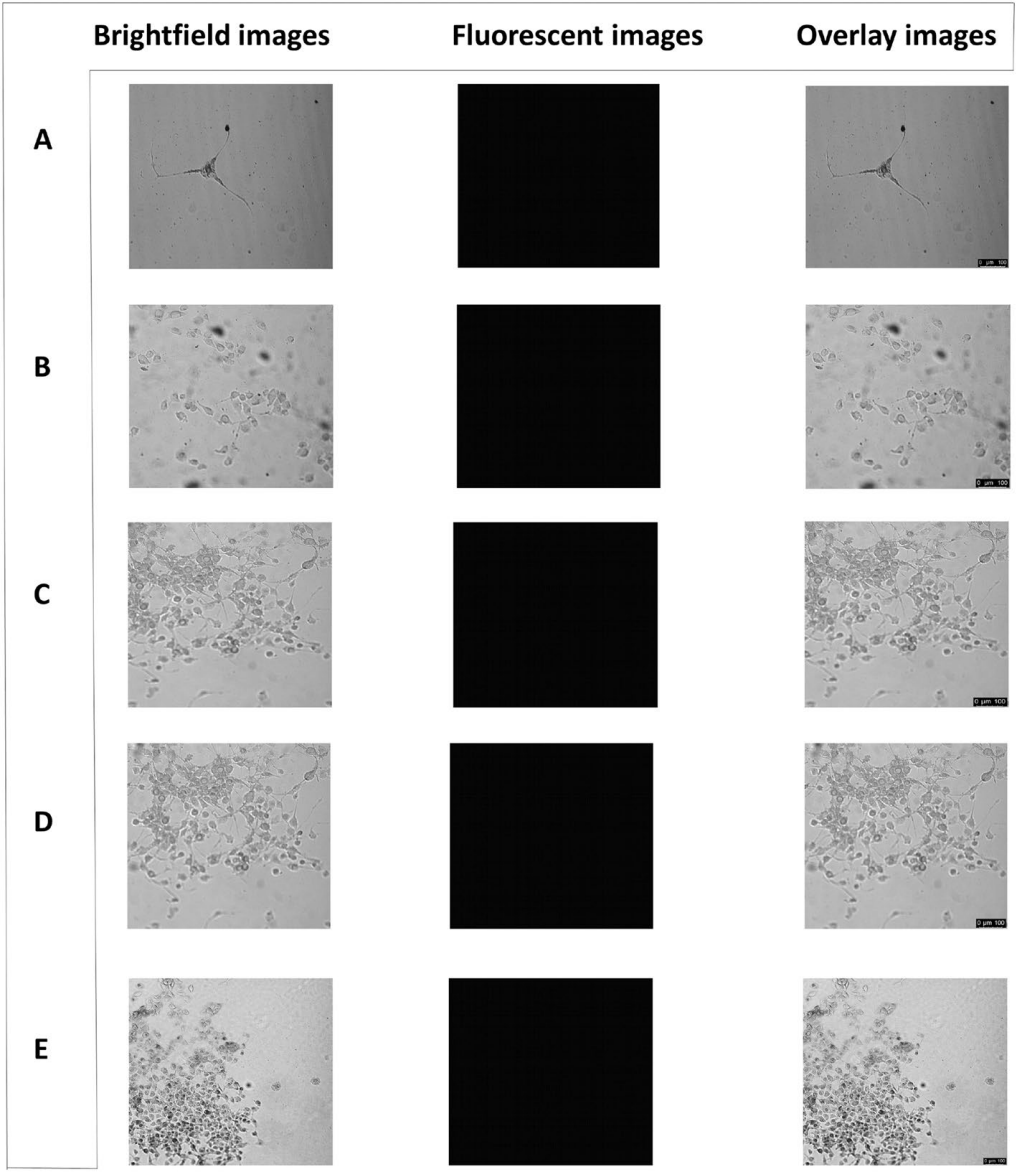
Confocal microscopy images of HepG2 cells after treatment with quantum dot and drug at pH = 7.4. (**A**) control untreated cells; (**B**) cells treated with 1/3rd IC50 value of quantum dot i.e., 18 ppm (**C**) cells treated with the drug and 18 ppm of quantum dot for 1 day; (**D**) cells treated with the drug and 18 ppm of quantum dot for 3 days; (**E**) cells treated with the drug and 18 ppm of quantum dot for 5 days. Brightfield images, fluorescent images, and overlay images are shown in the left, middle, and right panels respectively. Magnification: 200X. Zoom factor: 1.00. Scale bars are in the range of 0–100 µm.

To confirm that the quantum dot in question can only penetrate the human breast cancer cell line MDA-MB-231 and carry the drug along with it into the cells, the human liver cancer cell line HepG2 was treated with both the quantum dot and drug. The cellular uptake and release of the drug were confirmed by confocal laser scanning microscopy. The fluorescent property of the quantum dot was utilized to observe the effective endocytosis of the quantum dot. However, HepG2 cells treated with a quantum dot did not exhibit any fluorescence suggesting that the quantum dot could not invade the cells. As a result, the cells treated with both the quantum dot and drug over three different time points did not show any blue fluorescent signal as the quantum dot being impermeable to the HepG2 cells could not carry the drug inside the cells. The fluorescent images for all the treatment groups appeared black (Fig. 7).

The reason of least penetration efficacy of QDs in HepG2 cells may be due to the lowered adhesion of QDs in the cell membranes. The size, shape, surface chemistry, and subsequent internalization by the cells via energy-dependent pathways of the QDs are equally significant during the cell uptake process.

The drug release profile for QD showed that pH 7.4 (42% in 60 h) released substantially less drug than pH 5.0 (62% in 60 h) and pH 3.5 (80% in 60 h). Due to the ultrasmall ZnO QDs instantly dissolving within a few seconds when the pH was decreased, burst drug release was seen (Figure S3). The drug release profile of cell lysate at various pH levels presented here closely resembles the ZnO QD breakdown in mildly acidic conditions.

We have also checked the content of the final samples in the MDA-MB-231 cancer cells by taking the fluorescence measurements compared with the intensity of the bare QDs (Figure S4).

We have also performed densitometric analysis to quantitatively determine the fluorescence of QDs in MDA-MB-231 cells (pH = 7.4 and 3.5) and HepG2 cells (pH = 7.4) (Figure S5).

In order to demonstrate an anti-cancer drug delivery strategy against MDA-MB-231 cells, we used a pH-sensitive quantum dot-drug system. The drug was loaded onto the quantum dot by forming a complex with it. The drug was released under acidic conditions inside the breast cancer cells (MDA-MB-231) as compared to the liver cancer cells (HepG2) due to the disruption of the quantum dots. Studies using confocal microscopy demonstrated that the quantum dots in MDA-MB-231 cells were released intracellularly and disintegrated. Also, the MTT assay confirmed that the quantum dot carriers alone can reveal antitumor activity and favorably kill breast cancer cells. Hence, the apoptosis of cancerous cells is substantially improved by combining the drug with the quantum dot.

## Conclusion

In this study, we designed a biocompatible, nontoxic DETA-capped ZnO quantum dot to overcome MTX resistance on its resistant cancer cell MDA-MB-231. High-efficiency drug delivery of MTX to the MDA-MB-231 cancer cell, which is resistant to the medication, is made possible by this quantum dot. The efficient attachment of MTX to the QD is strongly corroborated by fluorescence and UV–Vis spectroscopy. Furthermore, a confocal microscopic study has also been conducted to examine the successful release of MTX on breast cancer cell MDA-MB-231. It has been established that QD successfully delivers MTX to breast cancer cell MDA-MB-231 at a higher rate on an acidic pH than it does at a physiological pH. Hence, it can be summarized that the quantum dots act as a pH-responsive nanocarrier to demonstrate significant anti-cancer activity, and thus the drug and quantum dots together execute synergistic cytotoxicity against breast cancer cells. Hence, the pH-responsive quantum dots-based delivery system becomes advantageous as a practical and distinctive drug delivery method that can improve the therapeutic outcome of chemotherapy.

## Experimental section

### Materials and methods

We bought Diethylenetriamine (DETA) and Zinc Acetate Dihydrate from Sigma-Aldrich Pvt. Ltd. Sodium Hydroxide (NaOH) was purchased from Himedia. The solvent in this instance is double-distilled (dd) water. The reagents for biological work like Foetal bovine serum (FBS), MTT (3-[4,5-dimethylthiazol-2-yl]-2,5-diphenyltetrazolium bromide) powder, DMSO, and Dulbecco's Modified Eagle's Medium (DMEM) were procured from Himedia Laboratory, India, while Mitomycin-C (Kyowa Hakko Kogyo Ltd., Japan) was acquired from Sisco Research Laboratories in India.

### Characterization

To comprehend the morphology of the QD, a transmission electron microscope (HR-TEM JEOL JEM 2100) was employed. On Malvern Instrument Ltd., DLS data were acquired. Using a Perkin Elmer Model LS 55 spectrophotometer, fluorescence spectra were captured. A SHIMADZU UV-3101PC spectrophotometer was used to record UV spectra On a Nexus TM 870 spectrophotometer, Fourier transforms infrared (FT-IR) measurements are collected. A confocal laser scanning microscope (Leica TCS SP8) was used to capture images of individual cells.

### Synthesis of amine (DETA) capped ZnO QD

First, 0.4 g of zinc acetate dihydrate was dissolved in 10 ml of double distilled water. Then add 1 ml DETA into the solution and stir vigorously. In a separate beaker, NaOH (0.2 g) solution was prepared. After that, add NaOH solution dropwise into the solution of zinc acetate dihydrate & DETA and stir it for 8 h. After centrifugation and vacuum drying, the amine-functionalized ZnO nanoparticles were obtained.

### Drug loading

To the as-prepared amine-capped ZnO QDs aqueous solution add 10^–3^ M MTX solution and stir for 5 min. Finally, the loading of MTX onto the amine-capped ZnO surface was confirmed by fluorescence and UV–Vis spectroscopy. The drug loading content was calculated as follows:

$$ {\text{drug loading content }}\left( \% \right) \, = \, \left( {{\text{weight of drug in nanoparticles}}/{\text{ weight of nanoparticles taken}}} \right) \, \times { 1}00 $$

### Anti-proliferative activity tests

The supporting information contains a detailed description of the anti-proliferative activity test protocol.

### MTT assay

The cytotoxic effect of the provided sample against MDA-MB-231 cell line has been analyzed in vitro by performing a cell viability assay or MTT assay. Mitomycin-C was used as the reference drug compound. The cells treated with mitomycin-C showed 50% survivability. The quantum dot exerted a cytotoxic effect against MDA-MB-231 cells in a dose-dependent manner and half-maximal inhibitory concentration, i.e., the IC_50_ value was found to be 36 ppm after 48 h of treatment. (See Supporting Information Figure S6).

Evaluation of the biological activity of the quantum dot reported herein showed IC_50_ value to be 36 ppm against MDA-MB-231 cells. The cytotoxic activity against MDA-MB-231 cells is dose-dependent. The quantum dot is a potent cytotoxic agent at a relatively low concentration against MDA-MB-231 breast cancer cells and may thus find applications in breast cancer therapy.

### Cellular uptake and confocal laser scanning microscopy (CLSM) tests

A thorough explanation of the cell line culture and the experimental setup for confocal laser scanning microscopy are both included in the supplementary data (Figure S7).

### Associated content

Binding constant calculation, UV–Vis absorption spectra of QD upon addition of MTX, Anti-proliferative activity tests, Cytotoxic effect of quantum dot against MDA-MB-231, Confocal laser scanning microscopy (CLSM) tests methods.

### Supplementary Information

Supplementary Information.

## Data Availability

All data supporting this study and its findings are available within the article and its Supplementary Information or from the corresponding authors upon request**.**

## References

[1] Chen S, Cai J, Zhang W, Zheng X, Hu S, Lu J, Xing J, Dong Y (2014). Proteomic identification of differentially expressed proteins associated with the multiple drug resistance in methotrexate-resistant human breast cancer cells. Int. J. Oncol..

[2] Bukowski K, Kciuk M, Kontek R (2020). Mechanisms of multidrug resistance in cancer chemotherapy. Int. J. Mol. Sci..

[3] Cai X, Luo Y, Zhang W, Du D, Lin Y (2016). pH-sensitive ZnO quantum dots-doxorubicin nanoparticles for lung cancer targeted drug delivery. ACS Appl. Mater. Interfaces.

[4] Lindgren M, Aizman RK, Saar K, Eiríksdóttir E, Jiang Y, Sassian M, Ostlund P, Hällbrink M, Langel U (2006). Overcoming methotrexate resistance in breast cancer tumour cells by the use of a new cell-penetrating peptide. BiochemPharmacol..

[5] Gorlick R, Goker E, Trippett T, Waltham M, Banerjee D, Bertino JR (1996). Intrinsic and acquired resistance to methotrexate in acute leukemia. N. Engl. J. Med..

[6] Bosson G (2003). Reduced folate carrier: biochemistry and molecular biology of the normal and methotrexate-resistant cell. Br. J. Biomed. Sci..

[7] Bertino JR, Göker E, Gorlick R, Li WW, Banerjee D (1996). Resistance mechanisms to methotrexate in tumors. Oncologist..

[8] Chen YH, Tsai CY, Huang PY (2007). Methotrexate conjugated to gold nanoparticles inhibits tumor growth in a syngeneic lung tumor model. Mol. Pharm..

[9] Issarachot O, Suksiriworapong J, Takano M (2014). Folic acidmodified methotrexate-conjugated PEGylated poly("-caprolactone) nanoparticles for targeted delivery. J Nanopart Res..

[10] Ji J, Wu D, Liu L (2012). Preparation, evaluation, and in vitro release of folic acid conjugated O-carboxymethyl chitosan nanoparticles loaded with methotrexate. J. Appl. Pol. Sci..

[11] Li M, Neoh KG, Wang R (2013). Methotrexate-conjugated and hyperbranched polyglycerol-grafted Fe_3_O_4_ magnetic nanoparticles for targeted anticancer effects. Eur. J. Pharm. Sci..

[12] Longgui Z, Ting S, Bin H, Zhongwei G (2014). Self-assembly polyrotaxanes nanoparticles as carriers for anticancer drug methotrexate delivery. Micro Nano Lett..

[13] Chittasupho C, Jaturanpinyo M, Mangmool S (2013). Pectin nanoparticle enhances cytotoxicity of methotrexate against hepG2 cells. Drug Deliv..

[14] Wei Y, Ma L, Zhang L, Xu X (2017). Noncovalent interaction-assisted drug delivery system with highly efficient uptake and release of paclitaxel for anticancer therapy. Int. J. Nanomed..

[15] Wu Z, Shah A, Patel N, Yuan X (2010). Development of methotrexate proline prodrug to overcome resistance by MDA-MB-231 cells. Bioorg Med Chem Lett..

[16] Ahar JM, Barar J, Alizadeh AM, Davaran S, Omidi Y, Rashidi MR (2016). Methotrexate-conjugated quantum dots: Synthesis, characterisation and cytotoxicity in drug resistant cancer cells. J Drug Target..

[17] Biju V, Mundayoor S, Omkumar RV (2010). Bioconjugated quantum dots for cancer research: Present status, prospects and remaining issues. Biotechnol Adv..

[18] Byers RJ, Hitchman ER (2011). Quantum dots brighten biological imaging. Prog Histochem Cytochem.

[19] Erogbogbo F, Yong KT, Hu R (2010). Biocompatible magnetofluorescent probes: Luminescent silicon quantum dots coupled with superparamagnetic iron(III) oxide. ACS Nano.

[20] Johari-Ahar M, Rashidi MR, Barar J (2015). An ultra-sensitive impedimetric immunosensor for detection of the serum oncomarker CA-125 in ovarian cancer patients. Nanoscale.

[21] Mashinchian O, Johari-Ahar M, Haemi B (2014). Impacts of quantum dots in molecular detection and bioimaging of cancer. BioImpacts.

[22] Qi L, Gao X (2008). Emerging application of quantum dots for drug delivery and therapy. Expert Opin Drug Deliv..

[23] Smith AM, Duan H, Mohs AM, Nie S (2008). Bioconjugated quantum dots for in vivo molecular and cellular imaging. Adv Drug Deliv Rev..

[24] Yuan Q, Hein S, Misra RDK (2010). New generation of chitosanencapsulated ZnO quantum dots loaded with drug: synthesis, characterization and in vitro drug delivery response. Acta Biomater..

[25] Hahn YB, Ahmad R, Tripathy N (2012). Chemical and biological sensors based on metal oxide nanostructures. Chem. Commun..

[26] Tripathy N, Ahmad R, Ko HA, Khang G, Hahn YB (2015). Enhanced anticancer potency using an acid-responsive ZnO-incorporated liposomal drug-delivery system. Nanoscale.

[27] Muharnmad F, Guo MY, Qi WX, Sun FX, Wang AF, Guo YJ, Zhu GS (2011). pH-triggered controlled drug release from mesoporous silica nanoparticles via intracelluar dissolution of ZnO nanolids. J. Am. Chem. Soc..

[28] Xia T, Kovochich M, Liong M, Zink JI, Nel AE (2008). Cationic polystyrene nanosphere toxicity depends on cell-specific endocytic and mitochondrial injury pathways. ACS Nano.

[29] Nel AE, Maedler L, Velegol D, Xia T, Hoek EMV, Somasundaran P, Klaessig F, Castranova V, Thompson M (2009). Understanding biophysicochemical interactions at the nano-bio interface. Nat. Mater..

[30] Cai W, Wang J, Chu C, Chen W, Wu C, Liu G (2018). Metal-organic framework-based stimuli-responsive systems for drug delivery. Adv. Sci..

[31] De D, Sahoo P (2022). The impact of MOFs in pH-dependent drug delivery systems: progress in the last decade. Dalton Trans..

[32] Kong B, Zhu A, Ding C, Zhao X, Li B, Tian Y (2012). Carbon dot-based inorganic-organic nanosystem for two-photon imaging and biosensing of pH variation in living cells and tissues. Adv. Mater..

[33] Yang Z, Xu M, Liu Y, He F, Gao F, Su Y, Wei H, Zhang Y (2014). Nitrogen-doped, carbon-rich, highly photoluminescent carbon dots from ammonium citrate. Nanoscale.

[34] Rong MC, Lin LP, Song XH, Wang YR, Zhong YX, Yan JW, Feng YF, Zeng XY, Chen X (2015). Fluorescence sensing of chromium (VI) and ascorbic acid using graphitic carbon nitride nanosheets as a fluorescent "switch". BiosensBioelectron..

[35] Uriarte D, Domini C, Garrido M (2019). New carbon dots based on glycerol and urea and itsapplication in the determination of tetracycline in urine samples. Talanta.

[36] Mallakpour S, Madani M (2012). Use of silane coupling agent for surface modification of zincoxide as inorganic filler and preparation of poly(amide–imide)/zinc oxide nanocompositecontaining phenylalanine moieties. Bull. Mater. Sci..

[37] Kayo OV, Jefferson B, Luiz FCDO, Jefferson LF, Marco AS (2017). Synthesis of multicolor photoluminescent carbon quantum dots functionalized with hydrocarbons of different chain lengths. New Carbon Mater..

[38] Huang Y, Liao W (2019). Hierarchical carbon material of n-doped carbon quantum dots in-situ formed on n-doped carbon nanotube for efficient oxygen reduction. Appl. Surf. Sci..

[39] Yan Z, Hong M, Dan Z, Shanshan Z, Xiaoming Y (2015). One-step synthesis of high quantum-yield and excitation-independent emission carbon dots for cell imaging. Mater. Lett..

[40] Stern O, Volmer M (1919). “über die Abklingzeit der Fluoreszenz. Z. Phys..

[41] Joseph RL (1999). Principles of Fluorescence Spectroscopy.

[42] Xu Z, Genfu Z, Xiaoping T, Xingcan Q, Ting Z, Jingwei G, Long Y, Xiaoguang X (2019). Nitrogen-doped carbon dots with high quantum yield for colorimetric and fluorometric detection of ferric ions and in a fluorescent ink. Microchim Acta.

[43] Muhammad F, Guo M, Guo Y, Qi W, Qu F, Sun F, Zhao H, Zhu G (2011). Acid degradable ZnO quantum dots as a platform for targeted delivery of an anticancer drug. J. Mater. Chem..

