# Supplementary material for: Efficient delivery of methotrexate to MDA-MB-231 breast cancer cells by a pH-responsive ZnO nanocarrier
Source: Sci Rep. 2023 Dec 11;13:21899. doi: 10.1038/s41598-023-49464-9 (PMC10713526; doi:10.1038/s41598-023-49464-9)
Supplement: Supplementary file 1 — Supplementary Information. [file 41598_2023_49464_MOESM1_ESM.docx]

**SUPPORTING INFORMATION**

**Efficient delivery of Methotrexate to MDA-MB-231 breast cancer cells by a pH-responsive ZnO nanocarrier**

Jiko Raut,†^a^ Olivia Sarkar,†^b^ Tanmoy Das,^b^ Santi M. Mandal,^c^ Ansuman Chattopadhyay,^b^ and Prithidipa Sahoo*^a^

^a^Department of Chemistry, Visva-Bharati University, Santiniketan-731235, India.

^b^ Department of Zoology, Visva-Bharati University, Santiniketan 731235, India.

^c^Central Research Facility, Indian Institute of Technology Kharagpur, 721302, India

*Correspondence to: Prithidipa Sahoo (Email: prithidipa.sahoo@visva-bharati.ac.in)

Number of Page: 12

Number of Figures: 7

1. **Binding constant calculation graph (Fluorescence method):**

**
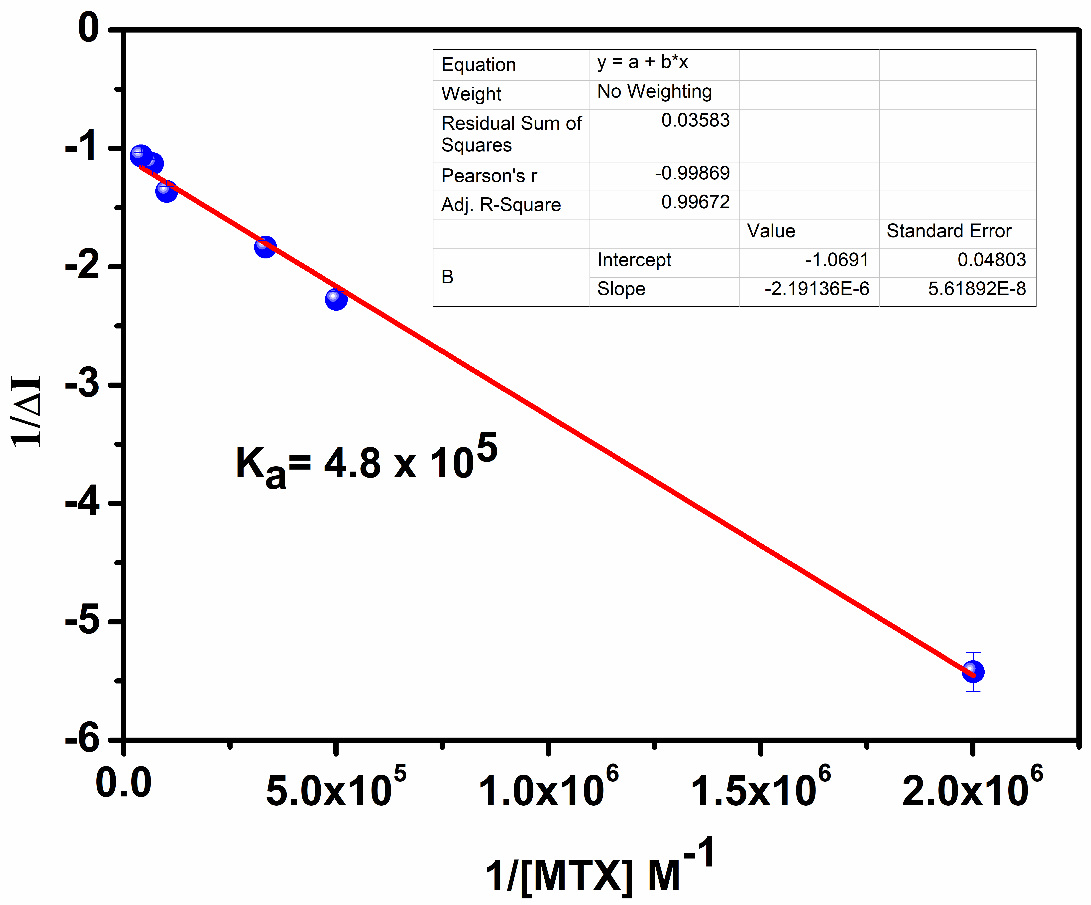
**

**Figure S1.** Linear regression analysis for the calculation of association constant value by fluorescence titration method.

The association constant (K_a_) of quantum dot for MTX was determined from the equation:
 K_a_ = intercept/slope. From the linear fit graph, we get intercept = 1.0691,
 slope = 2.19136 ×10^-6^. Thus, we get K_a_= (1.0691) / (2.19136 ×10^-6^) = 4.8 × 10^5^ M^-1^

1. **UV-Titration:**

**
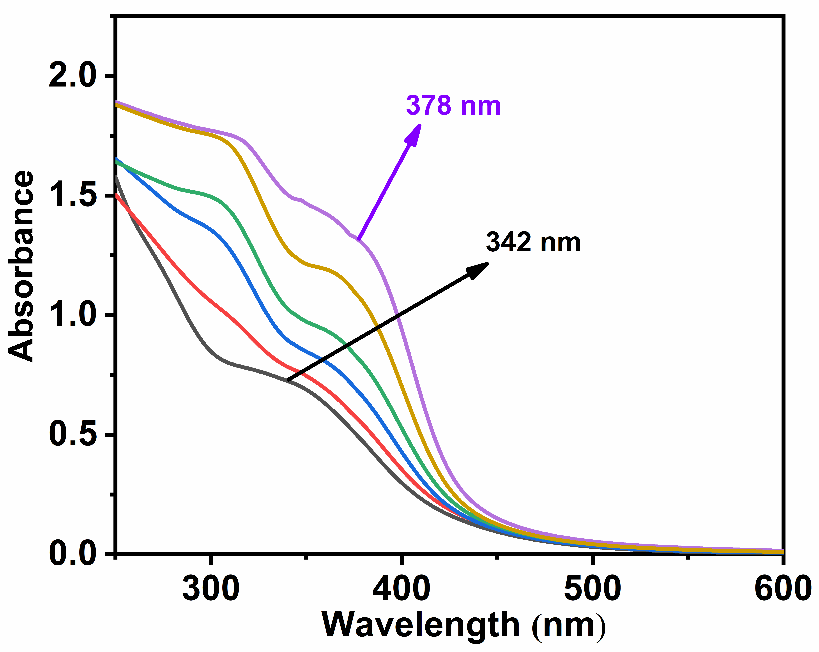
**

**Figure S2.** UV-vis absorption spectra of quantum dot upon addition of MTX (10^-3^ M).

1. **Drug release profile:**


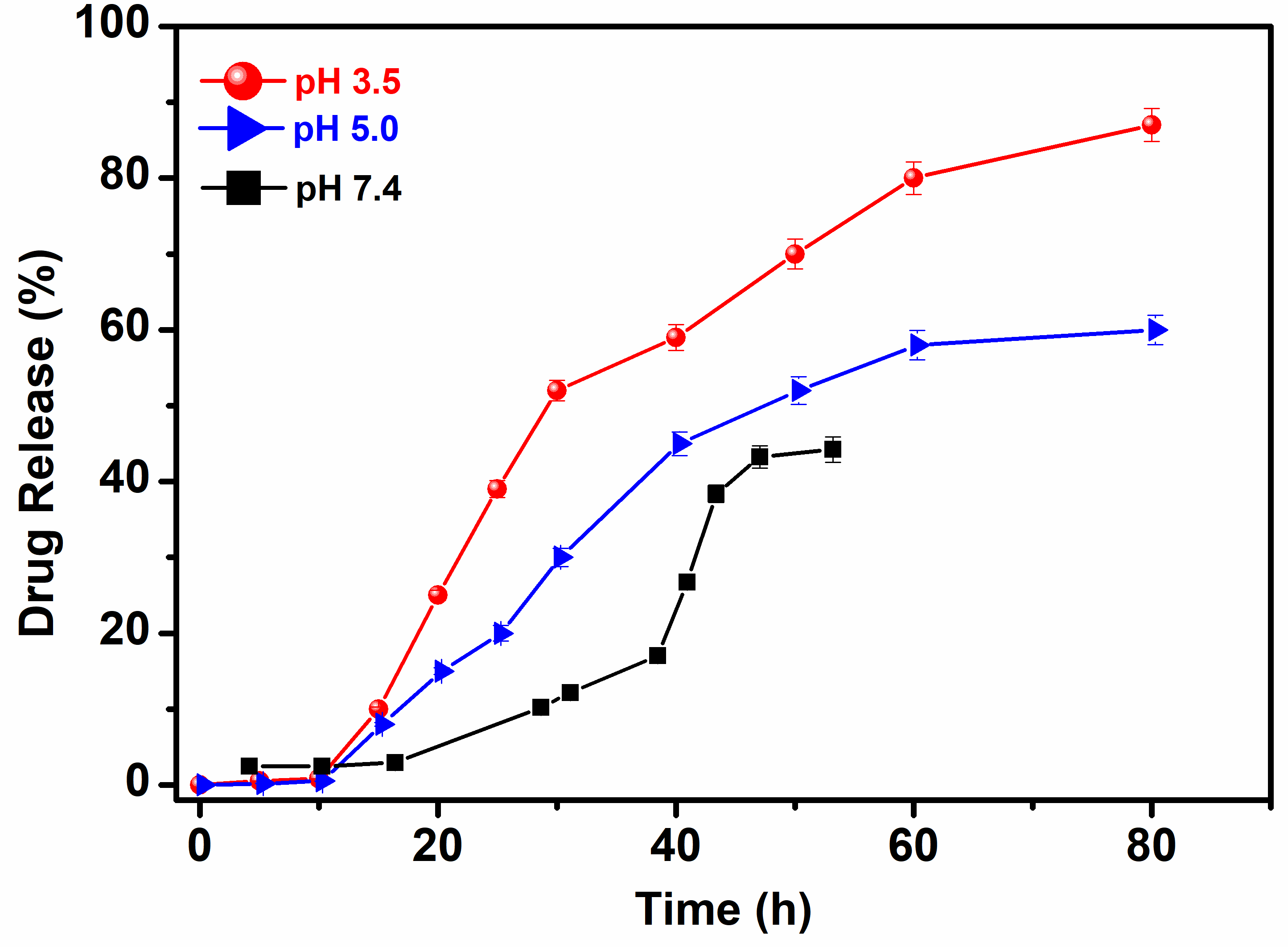


**Figure S3.** The drug release profile of the QDs at different pH environments.

1. **The concentration QDs after drug release:**


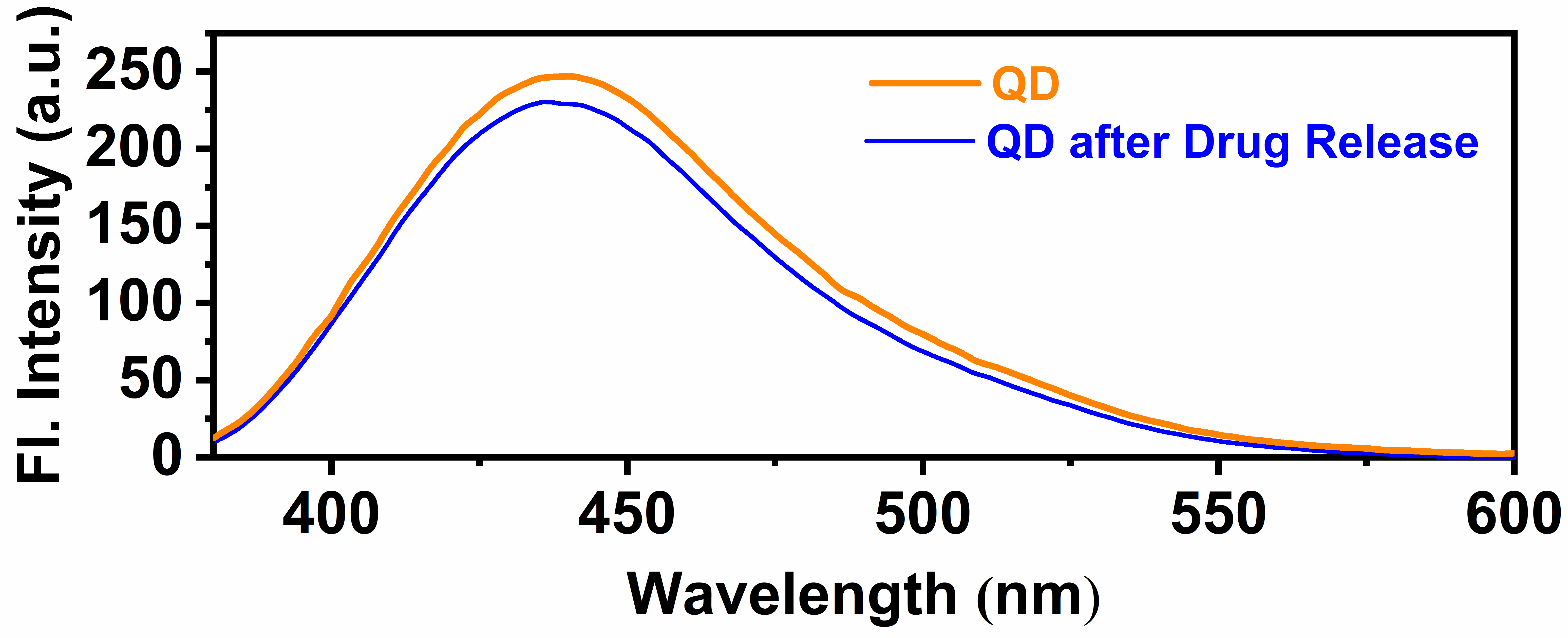


**Figure S4 a.** Fluorescence spectra of QD after drug release.


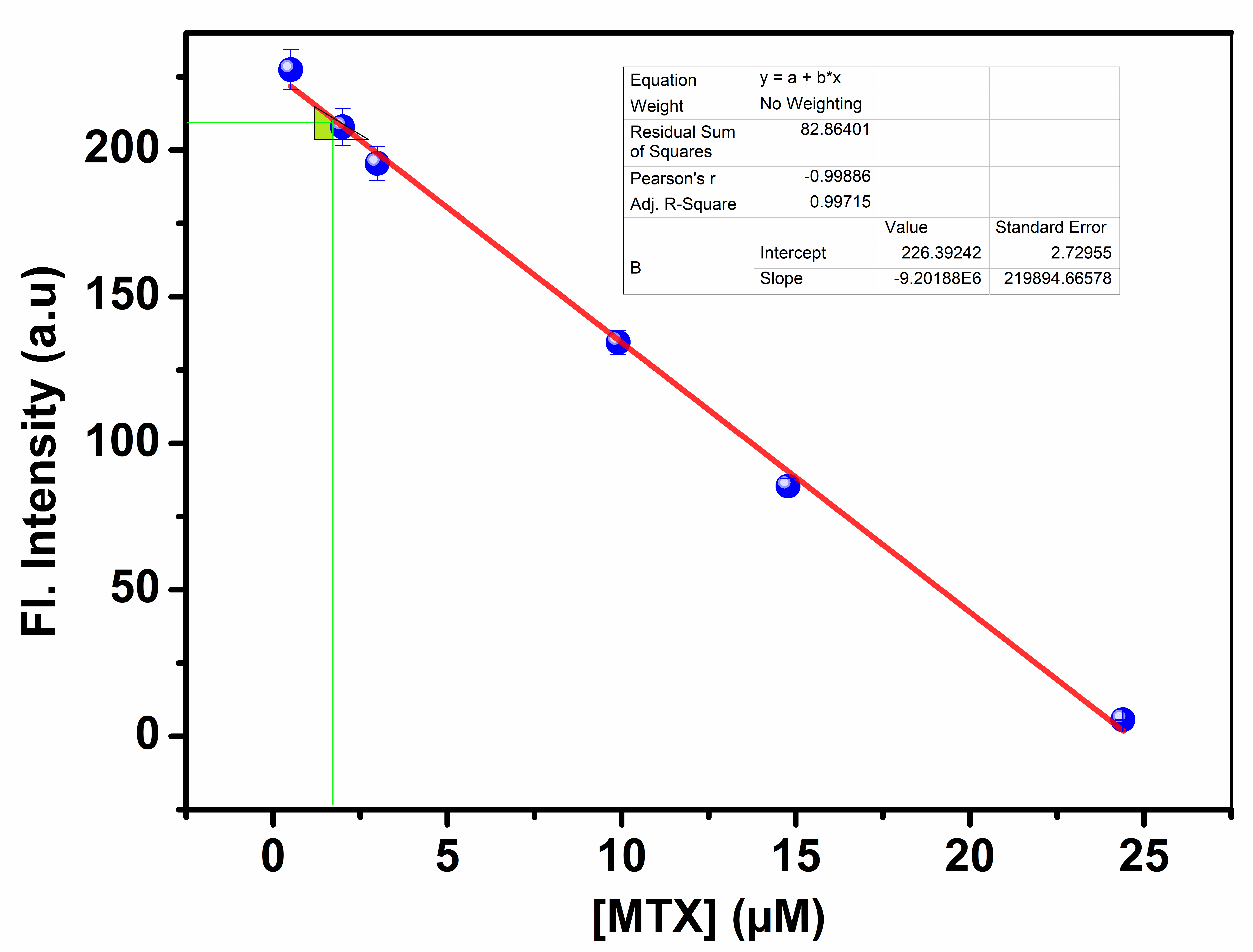


**Figure S4 b.** Estimation of the concentration (0.88 µM) of the QD after drug release from the standard fluorescence curve.

1. **Densitometric analysis:**


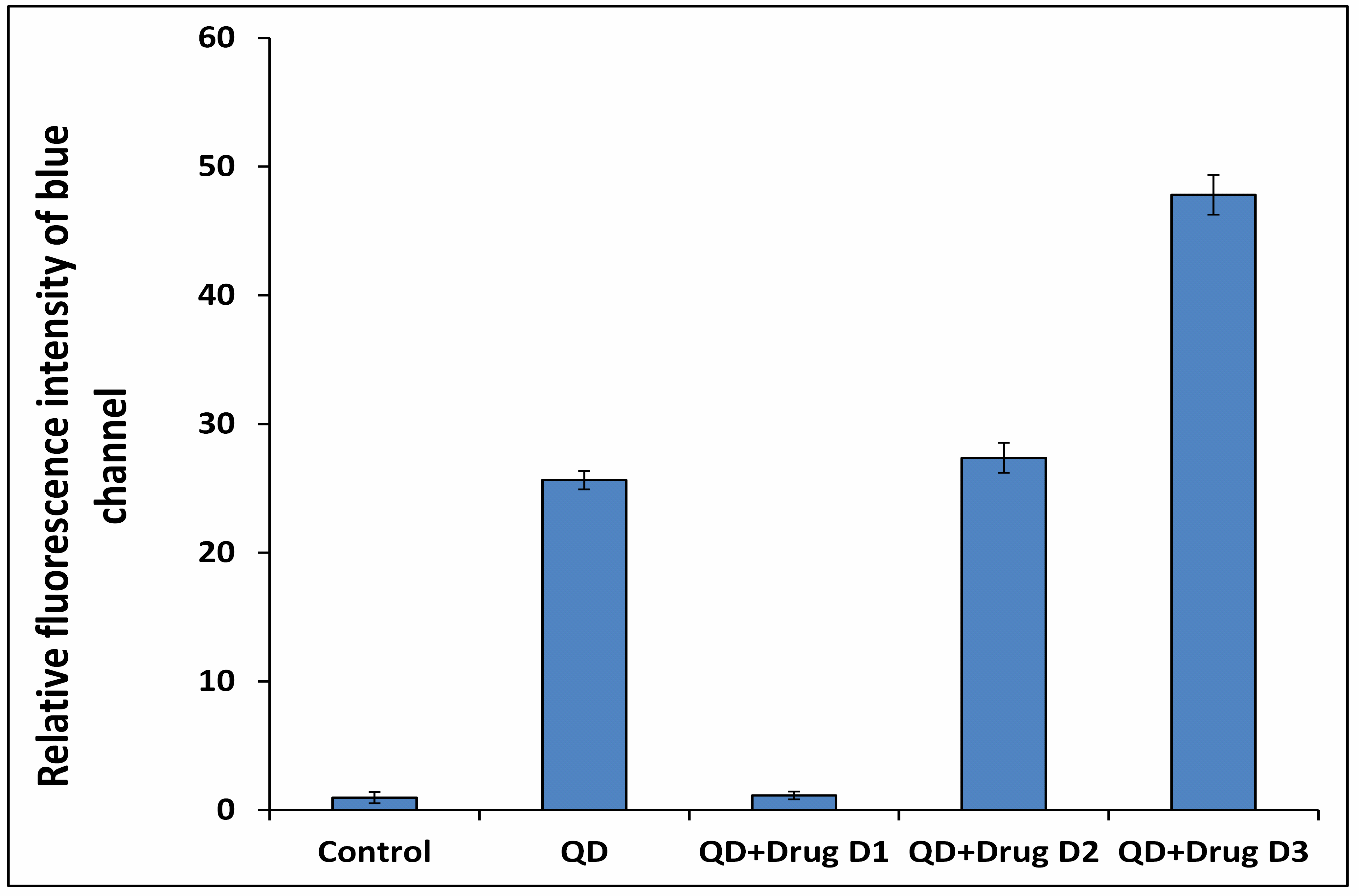


**Figure S5 a)** Relative fluorescence intensity (MDA-MB-231 at pH= 7.4) of the above-mentioned images was quantified using ImageJ v 1.46 software and graphed.


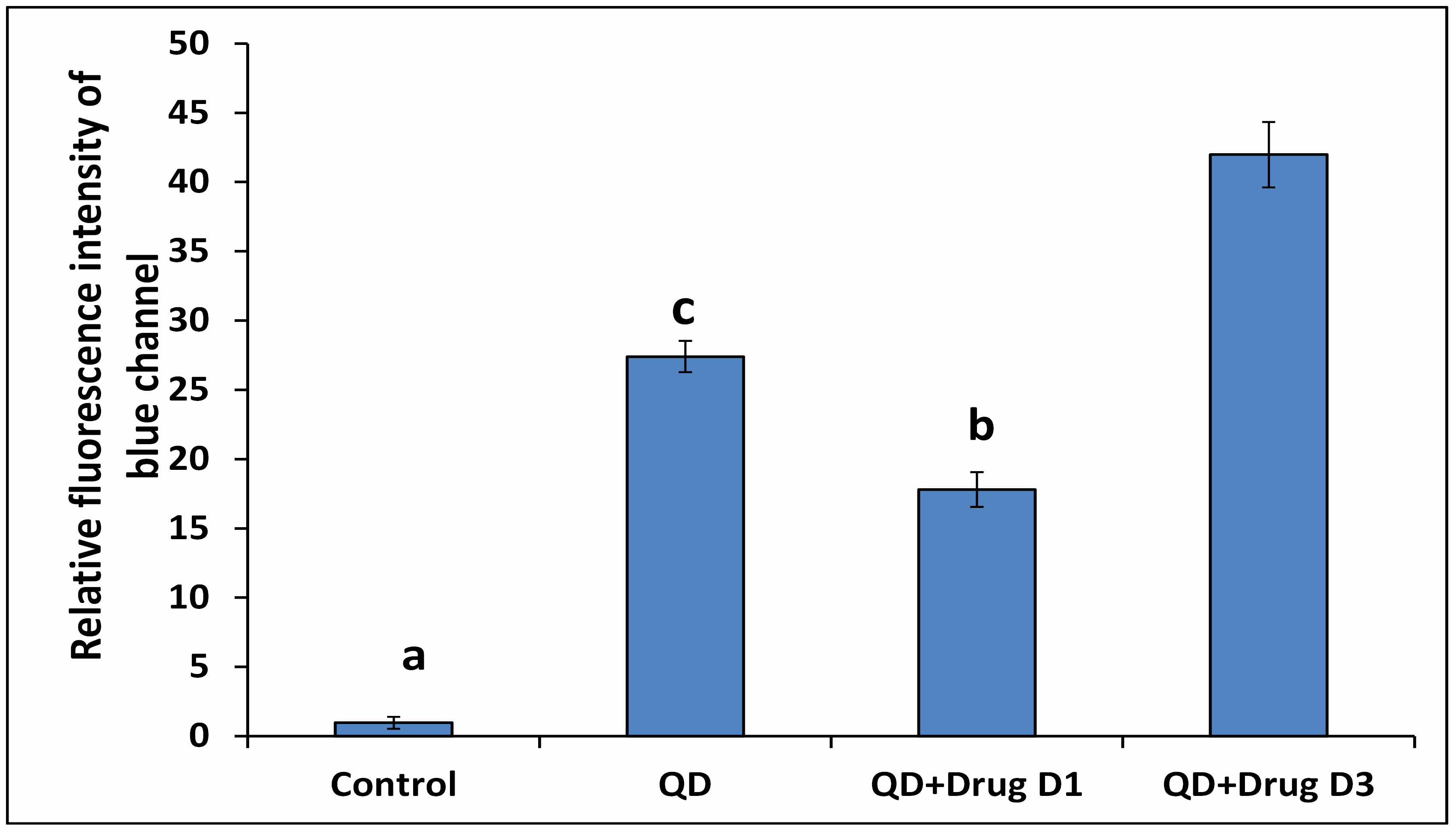


**Figure S5 b)** Relative fluorescence intensity (MDA-MB-231 at pH= 3.5) of the above-mentioned images was quantified using ImageJ v 1.46 software and graphed.


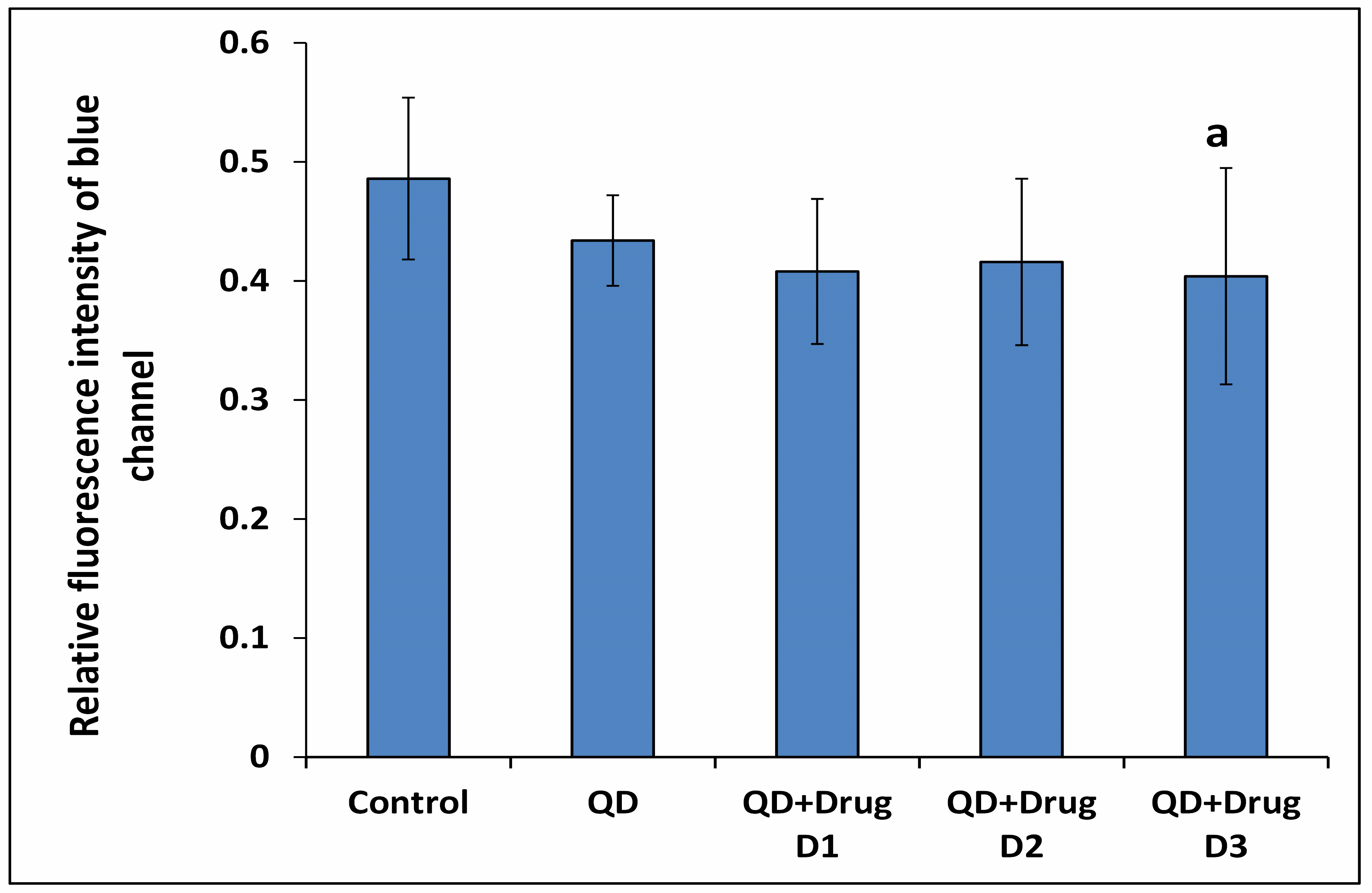


**Figure S5 c.** Relative fluorescence intensity (HepG2 at pH=7.4) of the above-mentioned images was quantified using ImageJ v 1.46 software and graphed.

1. **Anti-proliferative activity tests:**
2. **Cell line culture and experimental setup**

In brief, MDA-MB-231 was cultured in Dulbecco’s modified Eagle’s medium (DMEM) supplemented with 10% fetal bovine serum (FBS) and penicillin-streptomycin solution (1%) in a CO_2_ incubator (Thermo Fisher Scientific, USA) at 37°C and 5% humid atmosphere. The cells were grown till reaching 80% confluency and then plated for the subsequent tests.

1. **Anti-proliferative assays in MDA-MB-231 and determination of IC_50_ values**

The effect of quantum dot on MDA-MB-231 cell viability was assessed by MTT assay. MTT assay is a colorimetric test which is based on the conversion of the yellow tetrazolium salt to purple formazan crystals by the action of mitochondrial dehydrogenase (Mosmann, 1983). In brief, MDA-MB-231 was cultured in complete DMEM media supplemented with FBS (10%) and penicillin-streptomycin solution (1%) in CO_2_ incubator (Thermo Fisher Scientific, USA) at 37^°^C, 5% humid atmosphere. Cells were seeded in a 96 well plate at a density of 2 × 10^4^ cells mL^−1^ for this assay. After 24h, the cells were treated with different concentrations of quantum dot and cultured for another 48h. Untreated control cells and Mitomycin-C (50 μM)-treated cells were considered for negative and positive control respectively. Following which, 10 μL of MTT solution from 5 mg/mL MTT stock solution was used and applied. Purple coloured formazan crystals were dissolved in DMSO, and the optical density was measured at 595 nm in BIO-RAD Microplate Absorbance Reader iMark™ (USA). The OD value of the untreated control was calculated as equivalent to 100% living cells and cell survival (%) of quantum dot treated groups were estimated from respective OD values.

1. **Cytotoxic effect against MDA-MB-231:**

**A)**


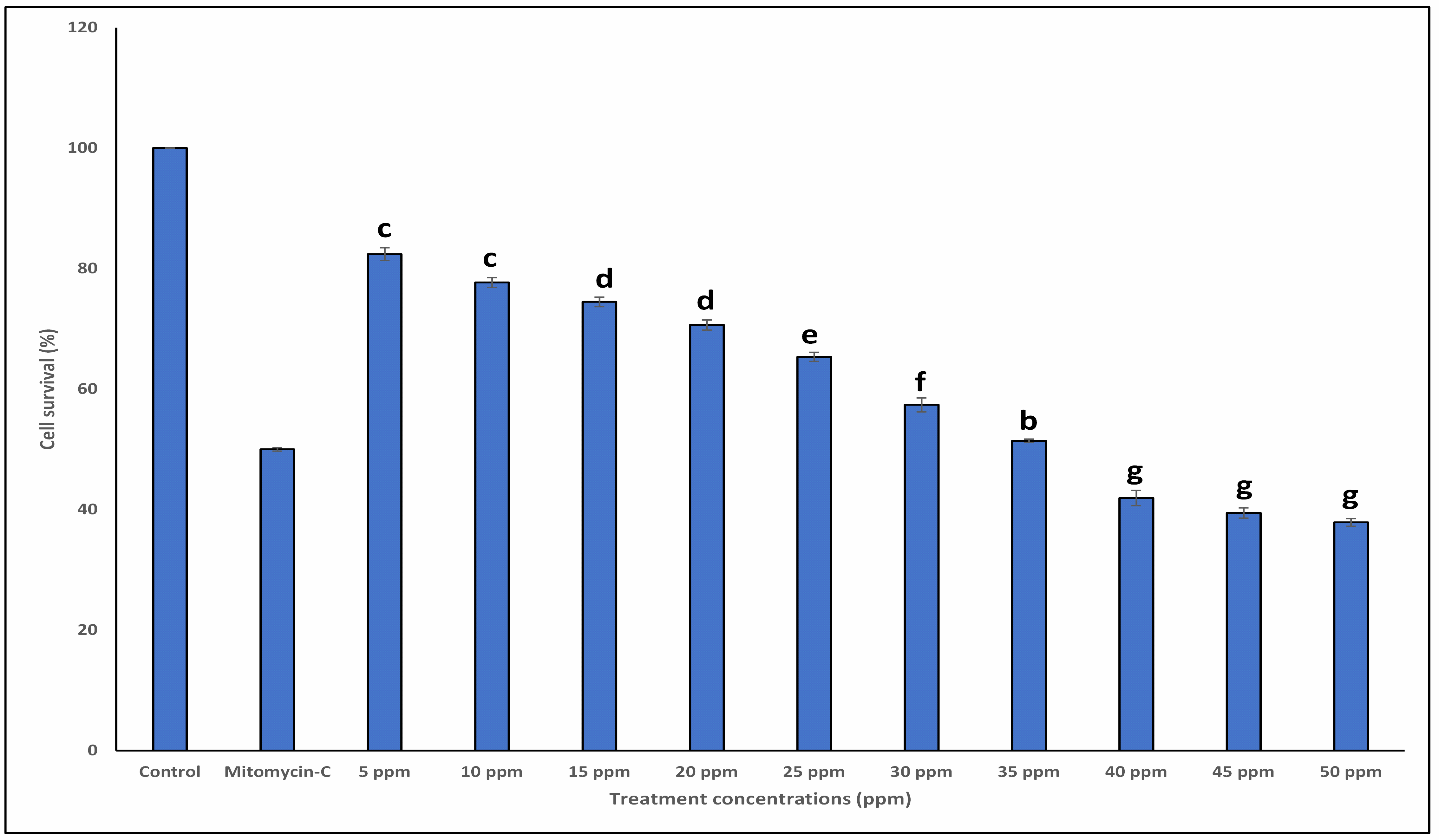


**Figure S6 a.** Survivability (%) of MDA-MB-231 cells upon exposure to increasing concentrations (5-50 ppm) of quantum dot for 48h. 50 μM Mitomycin-C was used as positive control. The half-maximal inhibitory concentration (IC_50_) was found to be 36 ppm after 48h of treatment.

Tests were performed in triplicate and the graphs represent their mean ± SEM value. Means with different letters are significantly different. Significance level α = 0.05.

**B)**


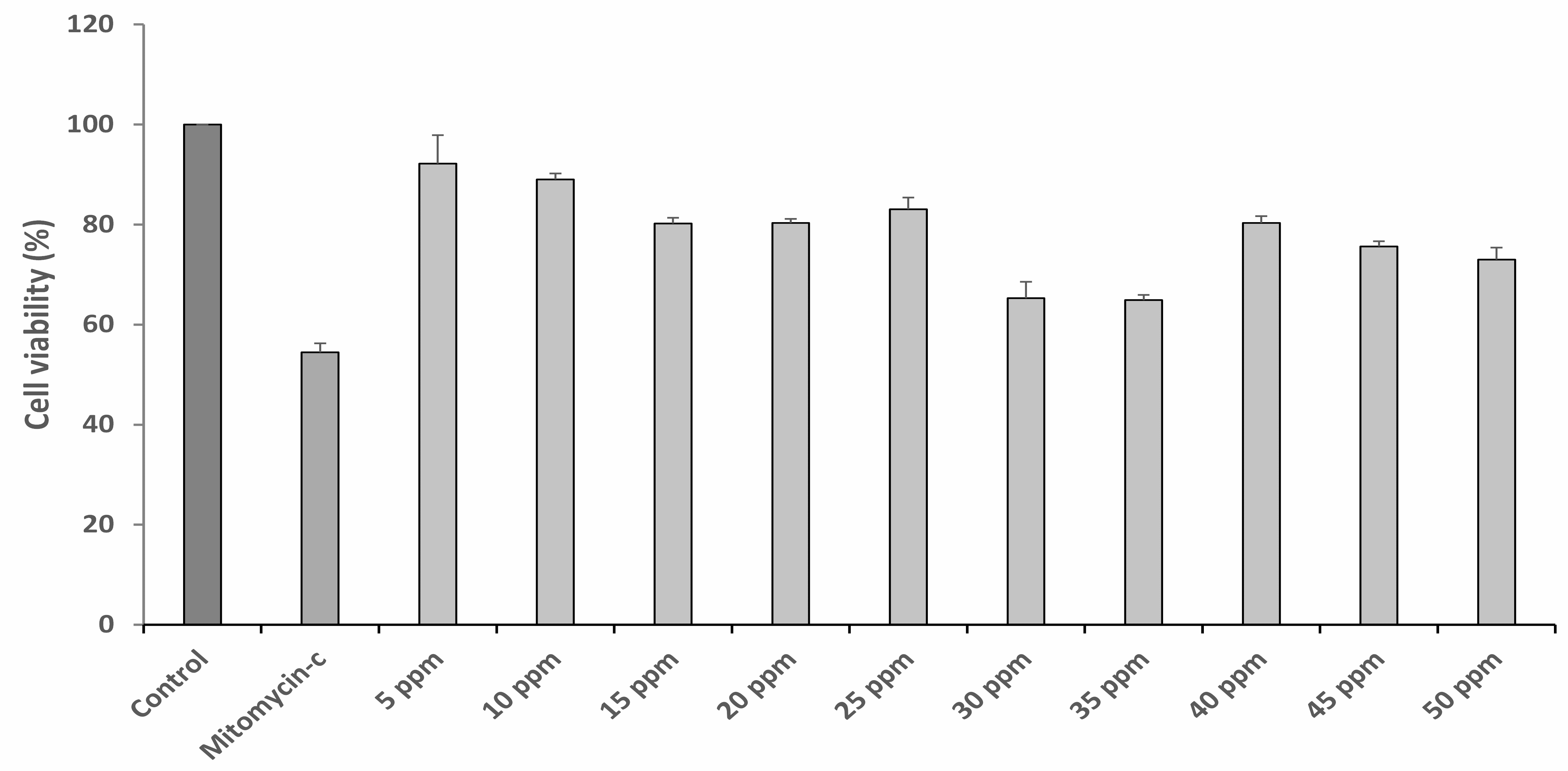


**Figure S6 b.** Cell viability (%) of MDA-MB-231 cells upon exposure to increasing concentrations (5-50 ppm) of drug for 48h. 50 μM Mitomycin-C was used as positive control. The half-maximal inhibitory concentration (IC_50_) was found to be 18.232 ppm after 48h of treatment.

**C)**


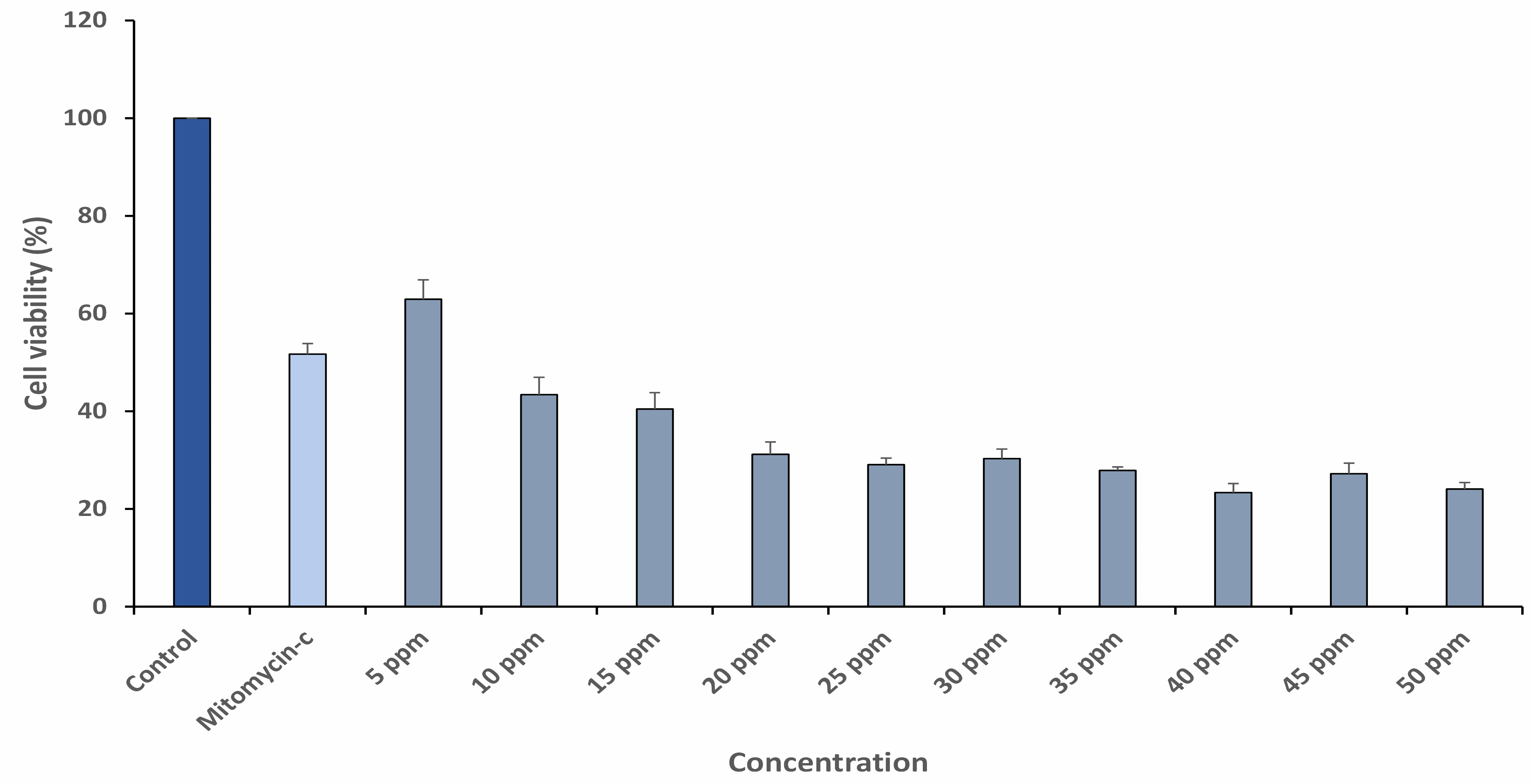


**Figure S6 c.** Cell viability (%) of MDA-MB-231 cells upon exposure to increasing concentrations (5-50 ppm) of drug with quantum dot for 48h. 50 μM Mitomycin-C was used as positive control. The half-maximal inhibitory concentration (IC50) was found to be 4.192 ppm after 48h of treatment.


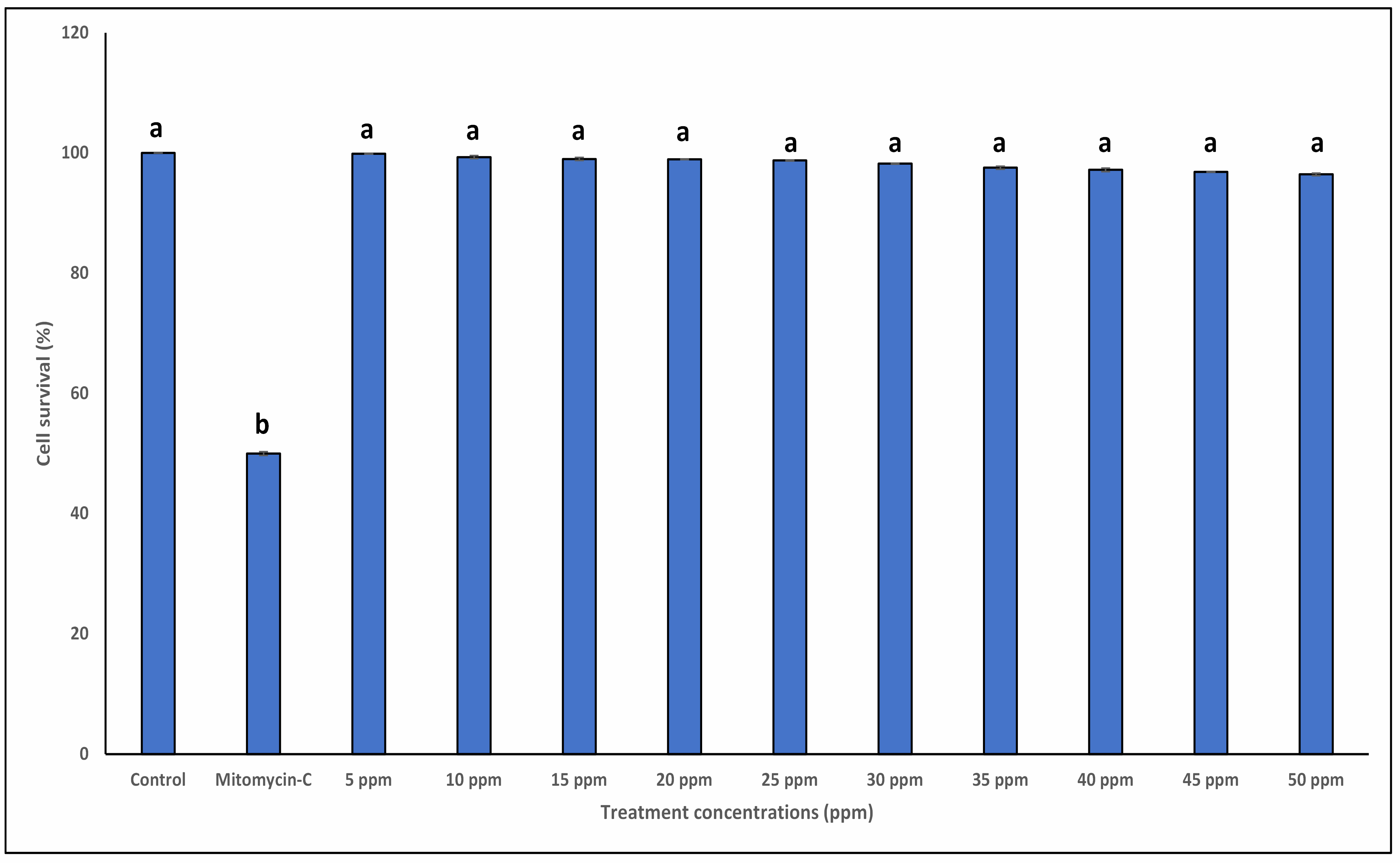


**Figure S6 d.** Survivability (%) of HepG2 cells upon exposure to increasing concentrations (5-50 ppm) of quantum dot for 48 h. 50 µM of Mitomycin-c was used as positive control.

Tests were performed in triplicate and the graphs represent their mean ± SEM value. Means with different letters are significantly different. Significance level α = 0.05.

1. **Methods:**

***Confocal laser scanning microscopy (CLSM) tests***

1. ***Cell line culture***

To check cellular internalization and intracellular release of the drug, the human breast cancer cell line MDA-MB-231 and the human liver cancer cell line HepG2 were cultured separately in 6-well cell culture plates with one piece of cover glass at the bottom of each chamber in incubation medium DMEM supplemented with 10% fetal bovine serum (FBS) and penicillin-streptomycin solution (1%) in a CO_2_ incubator (Thermo Fisher Scientific, USA) at 37°C and 5% humid atmosphere. Both HepG2 and MDA-MB-231 cell lines were procured from Dr. Surajit Sinha (Professor at Indian Association for the Cultivation of Science, Kolkata, West Bengal, India) which were purchased from NCCS, Pune, India. ^[1,2]^ The cells were grown until reaching 60% confluency and then used for the consequent experiments.

1. ***Cellular uptake and confocal laser scanning microscopy***

In the 6-well cell culture plates of both MDA-MB-231 cells and HepG2 cells, the cells of one well were used as untreated control i.e., not treated with either quantum dot or drug. The cells of one well were treated only with the drug. The cells of another well were treated with one-third of the concentration corresponding to the IC_50_ value of the quantum dot (i.e., one-third of 36 ppm = 12 ppm or 0.632 µL) for 1 day. In the remaining three wells, the quantum dot and drug were added into the incubation medium at the concentration of 12 ppm and cells from the three wells were used for three different time points i.e., 1 day, 3 days, and 5 days of post-treatment for imaging under confocal microscope. The physiological pH of 7.4 was maintained throughout the experiment. The experimental set up is depicted herein with a schematic diagram.


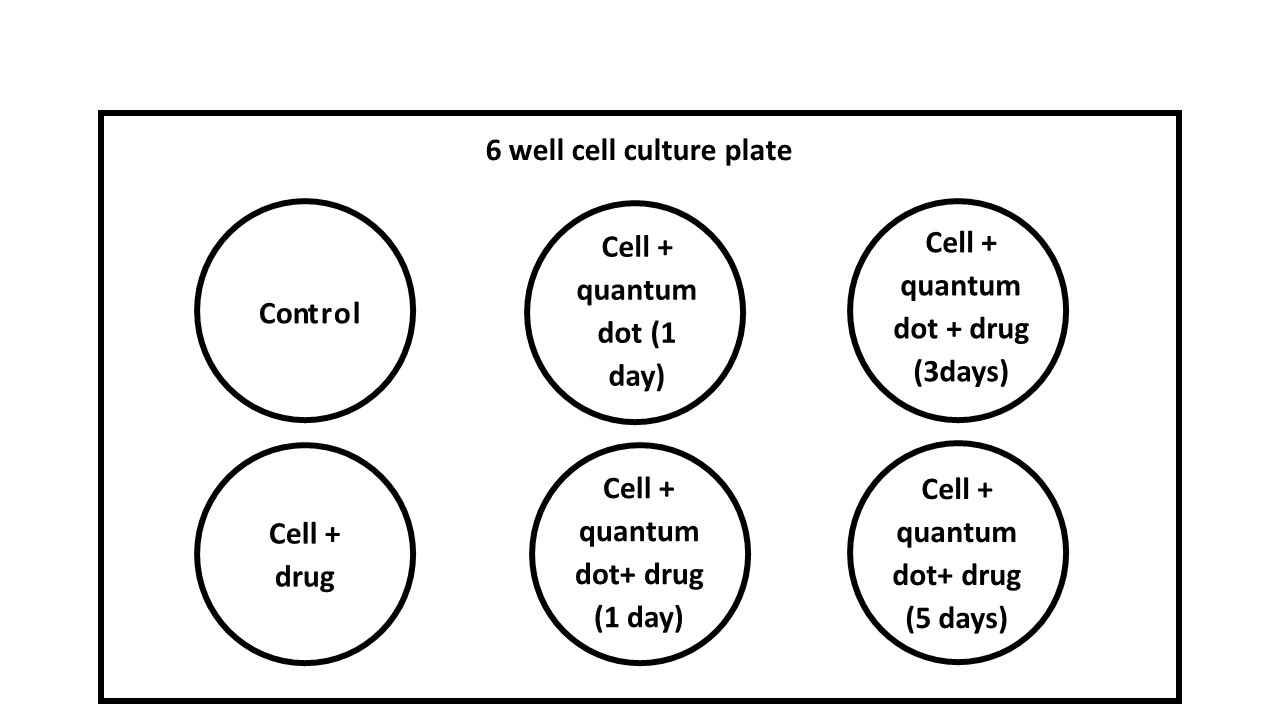
MDA-MB-231 cells were cultured in another 6-well cell culture plate where the entire set up was prepared to maintain an acidic pH of 3.5 (concentrated HCl was used at 0.1% concentration to the final volume of incubation medium of cells) to gauge the effect of pH on the dissociation of the drug from the quantum dot and eventually the dissolution of the quantum dot.

**Figure S7.** Experimental set up of culturing MDA-MB-231 cells and HepG2 cells in 6-well cell culture plates for treatment with quantum dot and drug.

After the completion of the treatment at different time points, the cells of each well were then washed thrice with PBS buffer (pH=7.4) and the cover glass from each well were immediately used and visualized for imaging under confocal laser scanning microscope (Leica TCS SP8). Blue channel filter excitation was set at 360 nm and emission at 440 nm. Images were taken with 20X magnification.

**References:**

1. Das, U.; Kundu, J.; Shaw, P.; Bose, C.; Ghosh, A.; Gupta, S.; ... & Sinha, S. Self-transfecting GMO-PMO chimera targeting Nanog enable gene silencing in vitro and suppresses tumor growth in 4T1 allografts in mouse. *Molecular Therapy-Nucleic Acids*, **2023**, 32, 203-228.
2. Sarkar, M. S.; Kar, D. A.; Shaw, D. P.; Chaudhary, D. S. K.; Keithellakpam, O.; Mukherjee, P. K.; ... & Sinha, S. Hydroalcoholic Standardized Root Extracts of Houttuynia Cordata (Thunb.) Promotes Apoptosis in Human Hepatocarcinoma Cell HepG2 Via GSK-3β/β-Catenin/PDL-1 Axis. *PDL-1 Axis*.
